# The feasibility of using photovoice as a loneliness intervention with older Myanmar migrants

**DOI:** 10.1111/nyas.15270

**Published:** 2025-01-28

**Authors:** Samia C. Akhter‐Khan, Chanyanut Wongfu, Nang Myat Pont Aein, Ben Lu, Matthew Prina, Sirinan Suwannaporn, Rosie Mayston, Khin Myo Wai

**Affiliations:** ^1^ Department of Global Health & Social Medicine King's College London London UK; ^2^ Department of Health Science, Institute of Public Health Mae Fah Luang University Chiang Rai Thailand; ^3^ Mae Sai District Chiang Rai Thailand; ^4^ Department of Aging & Epidemiology University of Newcastle Newcastle upon Tyne UK; ^5^ Faw Htoo Kaw Research Consultancy Yangon Myanmar

**Keywords:** Burma, coproduction, global South, participatory action research, social connection, social isolation, Southeast Asia

## Abstract

Loneliness has detrimental physical and mental health outcomes. To date, there are few studies on loneliness interventions in lower‐resource settings. Based on participatory action research methods that are theoretically informed by the social relationship expectations framework, we developed a loneliness intervention called *amanane* using the photovoice method with older Myanmar migrants in northern Thailand. The aim of our study was to test the feasibility and preliminary effectiveness of photovoice as an intervention for lonely older adults. Over 6 weeks, we coproduced 5 weekly workshops, individual interviews, and a photo exhibition with an older Myanmar migrant cofacilitator and nine participants (57–82 years old). The workshops focused on older people's care provisions. The qualitative evaluation entailed group discussions, interviews, and videos. Results indicated a perfect completion rate and high acceptability. Participants reported a reduction in loneliness due to opening up to each other through photography, feeling united despite cultural differences, and feeling valued by visitors attending the photo exhibition. Overall, photovoice may be a promising intervention for lonely older adults and has the potential to be tested in larger trials across diverse settings.

## Graphical abstract

Using the photovoice method, various elements of an intervention called *amanane* were identified as contributing factors to reduced loneliness among older Myanmar migrants in northern Thailand. Loneliness interventions that include high levels of participation, co‐production, and public engagement components seem feasible and promising. To test its effectiveness, feasibility, and sustainability, future studies will need to assess photovoice as an intervention for loneliness in larger randomized control trials with follow‐ups.

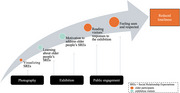

## INTRODUCTION

With the newly formed World Health Organization's (WHO) Commission on Social Connection,^1^ loneliness is recognized as a global public health priority given its detrimental impact on physical and mental health.^2^ Among the central aims of the commission is the identification and scaling up of effective solutions for loneliness. To date, there is limited evidence for cost‐effective interventions to reduce loneliness that can be scaled up globally.^3^, ^4^ As the recent WHO evidence‐gap map shows, loneliness interventions are especially sparse in lower‐ and middle‐income country (LMIC) settings.^4^ This gap is concerning, given that people around the world experience loneliness—with some LMICs showing higher prevalences than other high‐income countries^5^—and given that loneliness is closely related to contextual factors pertinent to LMICs, including poverty, stigma, and migration.^6^ While people at all ages may experience loneliness, studies suggest a U‐shaped trajectory across the lifespan, with loneliness being particularly prevalent in older age.^7^ Hence, there is a pressing need to identify loneliness interventions for older people in LMICs.

Although there may not be one single solution to reduce loneliness in later life, some reviews have highlighted key active ingredients for successful interventions, including active participation, productive engagement, community development, tailor‐made approaches, strong theoretical foundations, and variation in teaching and learning styles (which are often included in current interventions).^8^, ^9^, ^10^, ^11^ Most interventions that are built on theories apply theoretical frameworks that relate to loneliness or aging in a broader sense (e.g., social capital theory, socio‐emotional selectivity theory^12^), but do not address the core, cognitive definition of loneliness, that is, a subjective experience resulting from the gap between expected and actual social relationships.^13^ Our recently developed social relationship expectations (SREs) framework uses this core mechanism of loneliness to define six expectations that people have for their social relationships, that if unmet, may lead to feeling lonely.^14^ The SRE framework suggests that the expectations for generativity (e.g., providing meaningful contributions) and respect (e.g., feeling valued and included) are particularly relevant in later life and should, therefore, be integrated into solutions for older people's loneliness.

Inspired by the SRE frameworks’ focus on generativity and respect, we developed a complex intervention using photovoice, which is a method grounded in feminist theory and participatory action research^15^ that can give people the opportunity to meaningfully contribute and feel seen and valued. Photovoice is a collective visual research method combining photography with narrative data, discussions, interviews, and an action component (e.g., a photo exhibition).^15^ By combining various methods, photovoice provides access to multiple levels of explicit and tacit knowledge.^16^ A systematic review showed that photovoice can successfully be used with older people, is less reliant on cognitive functioning than other research methods, and can facilitate conversations around topics that would usually not be talked about.^17^

Although photovoice is typically not used as a mental health intervention—rather for purposes of describing participants’ perspectives, promoting critical dialogue, empowerment, creating social change, and evaluating programs^17^, ^18^, ^19^—studies have reported beneficial outcomes for participants’ mental health and well‐being. For example, photovoice has been shown to reduce levels of depression among rural female farmers in Nepal^20^ and reduce anxiety in a pilot study of college students living with a mental illness in the United States.^21^ Two recent photovoice studies suggested reduced loneliness or an increased sense of belonging as a result from their study; however, they did not provide detailed evidence for change.^22^, ^23^ Currently, there is, to the best of our knowledge, no study that has formally assessed photovoice as an intervention for reducing loneliness.

In the present study, instead of describing the photographs and messages that were created during the photovoice project, we aim to test the feasibility and preliminary effectiveness of photovoice as an intervention for lonely older migrants from Myanmar at the border region in Mae Sai, Thailand. The intervention was conducted with older people from Myanmar, as approximately one‐third of older adults reported feeling lonely during the past month in a national survey,^24^ and interventions have not yet been developed or tested among this population. Following the military coup in 2021 and ongoing conflict in the country, older people have reported a high prevalence of loneliness, along with other mental health issues.^25^ Due to Myanmar's political situation, people have been migrating to neighboring Thailand for decades, making up over 75% of the country's migrant population.^26^ Older adults from Myanmar in Thailand face additional challenges such as separation from family members, poor working conditions, language barriers, and discrimination, all factors that exacerbate the experience of loneliness.^26^, ^27^ Therefore, this pilot study focuses on evaluating contextual factors for realizing mechanisms of change, in line with the most recent Medical Research Council guidance on complex interventions^28^ and grounded in the SRE framework.^14^

## MATERIALS AND METHODS

Methods are reported according to the evaluation guidelines for social isolation and loneliness interventions by Schoenmakers et al.^11^ (Table S2).

### Setting

This study was conducted with Burmese‐speaking migrants from Myanmar living in a semi‐urban area in Mae Sai subdistrict, Chiang Rai province, Thailand. The area is located at the border to Tachileik, Shan State, Myanmar. Mae Sai subdistrict has an estimated population of 30,000 people. Most people living in the area are Thai, followed by Shan, Karen, and Burmese people. Whereas Thai people usually live in single houses, people from Myanmar live in apartments close to each other or smaller houses on the other side of the main street, close to a factory, where mostly people from Myanmar are employed. Mae Sai was chosen as the research site because of the prevalence of Myanmar migrant populations and the already established research connections between Thai authors (S.S. and C.W.) and local public health offices.

### Description of the intervention

#### Intervention components

*Amanane* a (အားမနာနဲ့, i.e., to not have anade အားနာတယ်) is an in‐person intervention consisting of five photovoice workshops, an exhibition, and individual interviews. The workshops were conducted weekly in the home of two of the participants living close to a factory, a place chosen as convenient and accessible by the participants. The sessions included an introduction to photography (e.g., technical use of camera, visual literacy/interpretation, captioning of photos), ethical issues, group discussions, soft skills (e.g., listening skills), and one‐on‐one interviews between the fourth and fifth session (see Supporting Information for the 5‐week workshop plan and interview topic guide). These procedures followed typical photovoice techniques described in previous studies.^16^, ^18^ The workshop plans for each week were created following the results from the previous workshop and discussions between the facilitators in order to be tailored to participants’ learning needs and potential challenges. At the end of each workshop, participants received a photography task that they were asked to complete by the next workshop. The first tasks were aimed at familiarizing participants with the camera (e.g., “Take a photo of something natural”) and became more complex by each session (e.g., “Photograph three things you value and three things you want to change”). The interviews were semi‐structured and followed a topic guide similar to the questions by Ronzi et al.,^16^ including the SHOWeD technique.^15^ In the fourth session, participants were asked whether they wanted to exhibit their photos and stories, which were selected by participants in the interviews, in a local photo exhibition that was subsequently curated and planned in the fifth session. Each session included dinner for all participants and a small compensation fee of 200 baht (approximately $5 USD) per workshop. All sessions were conducted in Burmese and audio‐recorded; group discussions, interviews, and the evaluation session were transcribed in Burmese and translated to English. Captions used in the photo exhibition were also translated to Thai to make the exhibition accessible to a broader audience. Field notes were taken throughout the project implementation.

#### Coproduction

The workshops were co‐facilitated by the first author (S.C.A.‐K.), who is a psychologist and PhD student from Germany and had lived in Myanmar before, where she had conducted research with older adults on loneliness for 1 year,^24^, ^29^ and a 60‐year‐old Karen migrant from Myanmar (B.L.) living in a neighboring village in Mae Sai. Both facilitators were women, spoke Burmese, had not conducted a photovoice study previously, and did not know the participants prior to the study. S.C.A.‐K. completed a training by PhotoVoice (https://PhotoVoice.org) prior to planning this study and met with B.L. several times to introduce her to using a camera and to discuss the workshop plan for each week. Having one co‐facilitator with prior experience of conducting qualitative research with older adults in Myanmar and migrants in Thailand^27^ and one co‐facilitator who was an older adult from Myanmar and spoke Burmese, Karen, and Thai was helpful in establishing trust with the participants as well as an understanding the environment, a key ingredient to successful participatory projects.^30^ Meeting older people to discuss the study procedure prior to the workshops and co‐facilitating the study with an older migrant also added an important coproduction component to this participatory action research project.

#### Program theory

*Amanane* is a complex intervention making use of a combination of different intervention strategies, including increasing opportunities for social interaction (e.g., meeting other older migrants from Myanmar and working on a project together) and leisure/skill development (e.g., learning about photography).^9^, ^31^ The intervention is based on our recently developed theory of loneliness, the SRE framework.^14^ According to the SRE framework, people feel lonely due to certain relationship expectations not being met, for example, proximity (availability of social contacts), support (feeling cared for), intimacy (feeling close, understood, and listened to), fun (sharing interests and enjoyable experiences), generativity (contributing meaningfully), and respect (feeling valued and actively included). Thus, interventions that aim to reduce loneliness should ideally target a wide range of these six relationship expectations in order for people not to experience a gap between their expected and actual social relationships (i.e., the cognitive definition of loneliness).^13^ *Amanane* has the potential to address all six SREs. As with any other group activity with the aim of social facilitation, our intervention can bring people together who would usually not meet frequently or talk to one another (proximity), where they can have discussions about personal issues and care for one another by establishing trust (support, intimacy), and identify and engage in shared interests (fun). Going beyond typical social facilitation interventions, *amanane* also addresses the other two expectations, generativity and respect, which according to the SRE framework become even more relevant toward the end of the lifespan. By visualizing older people's care contributions through photography and a community exhibition, participants may have the opportunity to not only contribute meaningfully to society (generativity), but also feel valued and seen for these contributions (respect).^32^, ^33^ Therefore, the thematic focus of the workshops was on care contributions and discussions about feeling valued as an older person, as providing care was described as a coping mechanism for loneliness in a study with Myanmar older adults.^29^ A more detailed description of the mechanisms by which the intervention is proposed to achieve reductions in loneliness, alongside hypotheses, can be found in a theory of change (Figure S1).

### Participant selection

#### Adaptation of the loneliness scale

To identify participants who were lonely, the De Jong Gierveld (DJG) loneliness short scale^34^ was chosen as it assesses both emotional and social dimensions of loneliness. Prior to implementation, the scale was culturally adapted to the Myanmar context. The final Burmese version of the DJG loneliness scale that was used to screen participants for inclusion in the photovoice project and the adaptation process can be found in Table S1.

#### Recruitment

To introduce the screening process and photovoice project to the Myanmar migrant community in Mae Sai, the first author (S.C.A.‐K.) met with a Burmese‐speaking female community health volunteer (CHV) and 10 older potential participants from a semi‐urban area in Mae Sai district, Chiang Rai. Information forms were distributed and questions about consent and the project plan were discussed. At first, participants were concerned about security issues because of the current political situation in the country and the wish to return to Myanmar one day. After reassuring the older people that the first author had lived in Myanmar before, is aware of the potential security issues, and will take care with keeping personal data private, participants expressed great interest in the study and reported being excited to participate as long as the study would not be about the political situation in the country. The screening process for administering the DJG short loneliness scale and consent forms was explained to the CHV and the first nine participants were screened together with the first author, making sure that participants understood the questions and provided answers independently. Participants also provided further information, including their age and available times to meet for the weekly workshops. Participants were eligible to participate when they felt lonely (score >2 according to the DJG loneliness scale), spoke Burmese, and were over 50 years old. Throughout January 2023, 13 participants were screened for inclusion. Among the 13 participants who were administered the loneliness scale during recruitment, 11 participants felt lonely (DJG loneliness scale score range: 5–10), two did not feel lonely. Of the 11 participants who felt lonely, nine decided to take part in the five workshops that took place on a weekly basis in January and February 2023 (*n* = 5 women; *n* = 4 men); two participants had time constraints, of whom one dropped out after attending the introductory workshop (Figure S2). Although a sample size of nine participants may seem small, this size is typical for photovoice studies.^18^ Participants’ ages ranged from 53 to 82, with an average age of 67 years. Among the nine participants, six were Karen and three were Burmese. The weekly workshops lasted between 1 and 2.5 hours. The study followed ethical principles of informed consent, was in line with the Declaration of Helsinki, and was approved by the institutional review board at King's College London (reference number: HR/DP‐21/22‐28513), the local Health Promotion Office in Mae Sai, and the head of the subdistrict where the study took place.

### Feasibility

In the focus group discussion, the acceptability, benefits, and challenges throughout the project participation were evaluated, as well as any advice for future project implementation. Another indicator of feasibility, and one of the main active ingredients of *amanane*, is active participation. Thus, we were interested in the levels of participation at each stage (e.g., to what extent it was possible to let participants take over control of the research process).^18^ According to Wang and colleagues’^35^ photovoice case study in Yunnan, there are four different levels of participation that a project can be situated in, ranging from contractual (i.e., researcher has control over the process) to collegiate (i.e., participants have control over the process). Thus, certain stages of any participatory action research study can be approached and conducted with different levels of participation depending on who has control over which project stage (i.e., researcher or local people). Field notes helped reflect on the levels of participation for each stage.

#### Changes to implementation

While there was excellent adherence to the planned length and frequency of the intervention (5 weekly workshops, exhibition, and interview), there were two adjustments to the project implementation. First, the planned content of each workshop could not be implemented exactly as planned because the exercises needed to be adjusted to the participants’ needs after every session (by S.C.A.‐K., B.L., R.M., and K.M.W.), which is typical for participatory research projects. Second, the method for captioning photos had to be altered. Initially, the idea for captioning the photos was to let participants write their own captions. However, several participants had vision problems and could thus not read or write captions, which is why the audio recordings of each group discussion were transcribed before the next workshop so that participants could match the transcripts with the photos (with the assistance of participants who could read). These two adjustments made the project more time‐intensive between the sessions but were necessary to tailor the intervention to participants’ individual needs.

### Preliminary evaluation

#### Individual outcomes

The final session following the exhibition was used as an evaluation session for the intervention using a focus group discussion (i.e., classified as “SIL intervention category I”^11^). Participants were not informed about the aim of photovoice as a potential intervention to reduce loneliness, and loneliness was not discussed prior to the evaluation session (unless it naturally occurred in the photo discussion). Quantitative analyses were not applied due to the small sample size and the aim of the project (i.e., focus on feasibility).

Results were triangulated from the focus group discussion, the individual interview, and field notes. As qualitative evaluation outcomes, participants were encouraged to give feedback and discuss their experience around participation, social relationships (e.g., changes in loneliness), perceptions in the wider community, and knowledge coproduction and sharing in a group discussion following the local exhibition. Participants’ perceived mechanisms underlying reductions in loneliness were analyzed and discussed between authors, while the relationship to the theoretical model underlying this study (i.e., the six SREs) was explored.

#### Community outcomes

As a secondary outcome, people's knowledge and attitudes about older people's contributions were assessed using a public engagement component during the exhibition. Stakeholders, including public health officers, students, lecturers, and community members who attended the exhibition were asked to anonymously answer written open questions that were provided on postcards in Burmese and Thai (e.g., what they have learned from the exhibition and how their attitudes have changed toward older people from Myanmar). Additionally, we recorded responses from participants and visitors at the exhibition to create a video about the intervention. Qualitative data were transcribed in Burmese, translated to English, analyzed using inductive thematic analysis, and coded in *NVivo* by S.C.A.‐K.^36^ After initial themes were identified, the pathways to reduced loneliness described by participants were compared with the six SREs.

## RESULTS

The evaluation of the feasibility and preliminary effectiveness of *amanane* as a loneliness intervention is presented in the following themes: individual outcomes, community outcomes, and implementation challenges. Table 1 illustrates the themes and categories, and how the themes were derived from multiple data sources.

**TABLE 1:** Overview of themes and data sources.

Themes	Categories	Data source
Individual outcomes	Levels of participation Participatory experience Changes in loneliness	Field notes Focus group evaluation Video material from participants Pre–post assessment of loneliness scale
Community outcomes	Positive response to the exhibition Improved understanding of older people Motivation to improve older people's lives	Postcards from the exhibition Video material from visitors at the exhibition
Implementation challenges	Security concerns Technical issues, time, and compensation Vision and language barriers Continuation of project	Field notes Focus group evaluation

### Individual outcomes

#### Levels of participation

**FIGURE 1:**
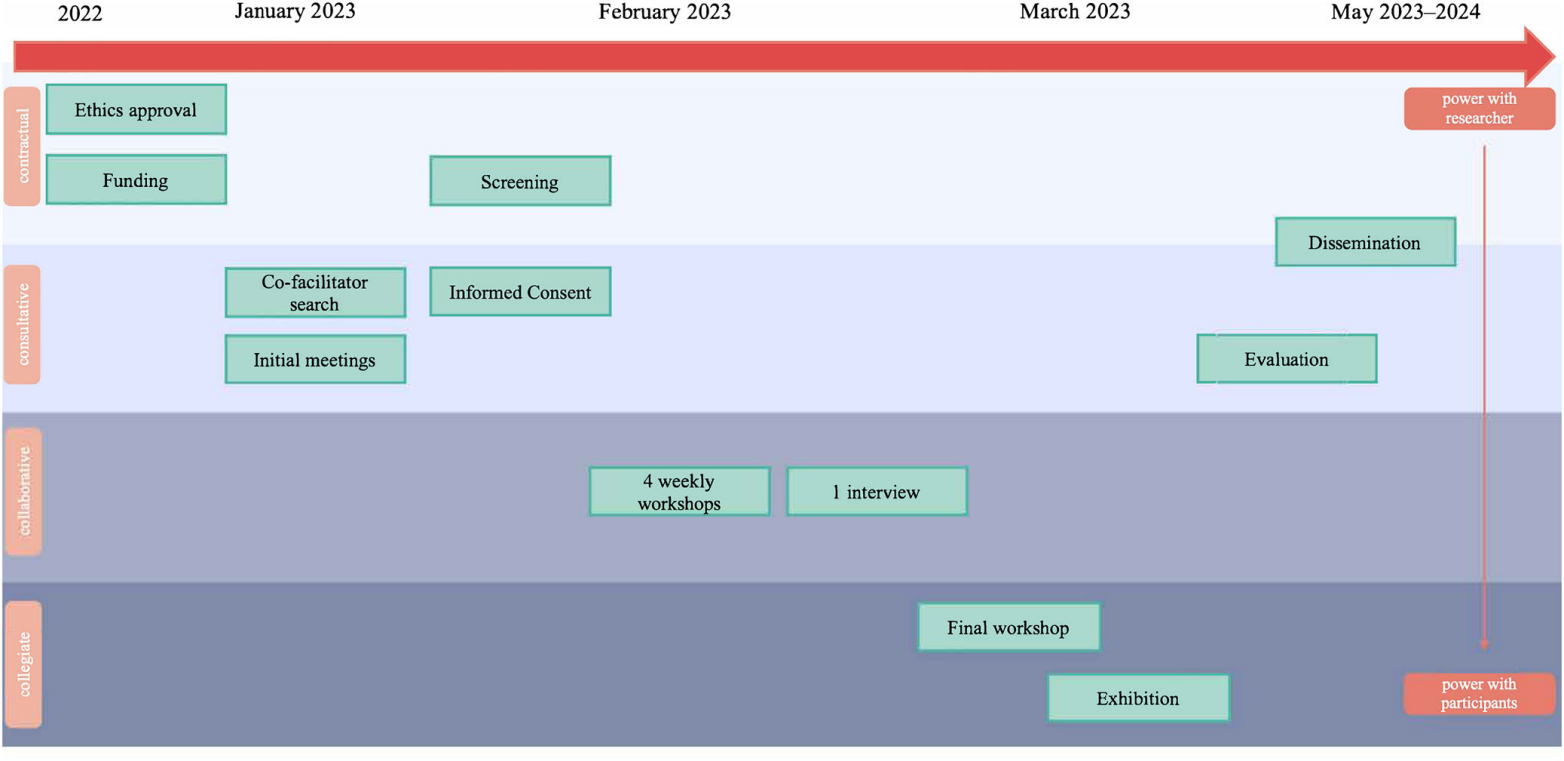
Intervention timeline with levels of participation. The different intervention components—ranging from design, implementation, and dissemination—are allocated to different levels of participation: contractual (people agree to take part in research), consultative (people are asked for their opinion), collaborative (researchers and local people work together on projects initiated by the researcher), and collegiate (researchers and local people work together as colleagues and local people have control over the process) (see Wang et al.^35^).

Figure 1 illustrates the project timeline with the intervention components of *amanane* and different levels of participation throughout the project. Most of the interventions’ components were implemented with high levels of participant engagement (between collaborative and collegiate). Participants had control over the entire process of the design and implementation of the project output (i.e., collegiate) by deciding to exhibit the photos in a local exhibition called “What is Care?” in March 2023. The exhibition was also shown at King's College London in May 2023, and can be viewed online at the Revaluing Care in the Global Economy Network by Duke University.^37^ However, there were certain stages of the project where participation was limited, and researchers solely decided about and implemented these parts. The intervention was part of a PhD project that had to be designed with limited time and budget. Thus, the funding and ethics approval application was designed by the researchers (from Germany, the United Kingdom, Thailand, and Myanmar) before any contact with local older people in northern Thailand was made (i.e., contractual). Furthermore, participants did not know about the aim of the project (as an intervention to reduce loneliness) and were recruited based on their levels of loneliness by the researchers, thus, were only fully informed and consulted in the evaluation phase. Another obstacle to participant engagement pertaining to the dissemination phase was the language barrier, as no participant spoke English and could, therefore, not contribute to writing journal articles. Nevertheless, co‐facilitator B.L. who was an older migrant from Myanmar was included as an author in this manuscript given her substantial contributions throughout the project implementation and evaluation.

#### Participatory experience

All nine participants attended each workshop, exhibition, and evaluation session. The workshops were perceived as enjoyable and a novel learning opportunity for older people, showing very good acceptability.

No one was giving excuses like ‘I can't leave home as there is no guardian at home or I have business to take care of.’ (…) We all are eager to join the workshop.

Participants felt cognitively stimulated by the workshops and learning photography skills. Some described their experience as being a student again.

We learned to live happily, to be united, to care for each other and have religious discussions. You feel like you are back to student life. (…) I started thinking about what kind of questions you will have for me next week.

Many participants described an initial worry about taking part in the project, as they were unsure as to whether they could manage to take photos themselves. This worry subsided quickly and made participants feel like they accomplished a small milestone in later life (e.g., “I was reluctant to take part in the project because I don't even know how to take a photo. Now I can do it well. Do you see?”).

#### Changes in loneliness

All participants were convinced that the intervention could reduce loneliness. Table 2 illustrates how different mechanisms led to reductions in loneliness, while mapping onto the six SREs. Before the project, participants described their relationships to each other as superficial acquaintances who did not know each other well and only paid attention to their differences (e.g., coming from different regions in Myanmar). Thus, they would only follow social norms and politely greet each other when meeting on the street. After participating in *amanane*, participants argued that they held great affection for one another, felt united from now on, and felt safe and strong by being surrounded by friends.

I opened up about my family, like, this and that happened. Then, half of the stress got off my chest when I got back home. I don't dare to talk to complete strangers though. You [pointing to another participant] and I are from the same area of the country. But I did not know you. (…) We met here only when the project started.

We are living together, like a family. (…) It's friendship for forever. This friendship is going to last for many more years to come.

**TABLE 2:** Changes in loneliness identified in the group discussion.

Mechanisms of change in loneliness	Participants’ quote	Social relationship expectations
Group photography helped overcoming differences	“Yes, we had little acquaintance. We were unapproachable to one another in the past because we are from different regions. We just behaved well for social customs. When this project came to take place, our acquaintance began to blossom. We are busy with the camera and shooting. Eager to take photos of each other.”	Proximity
Photography facilitated opening up to others	“I'm not lonely any longer because I have companions to unburden my feelings. We were less likely to open our hearts before we became close.”	Intimacy
Group activities facilitated caring friendships	“We are getting along now. We look after each other in every condition. For instance: we asked Ma Phyu* to go and see Ma Nway* when she was not feeling well. Previously, we were not likely to pay a visit to someone or accompany each other. Now, we are encouraging each other, sharing, and chatting all the time.”	Support
Participating in the study had benefits for others	“When the neighbors asked about camerawork and I showed the photo of a puppy (…) they were curious about my work and approached me to take their photos. In this way, I showed them how to shoot and they learned from me. Now, they have the experience of shooting.”	Generativity
The exhibition facilitated feeling valued and respected	“We know how to take care of children, care about household duties. We do everything, beginning with cooking. But we are not being paid for these jobs. When children came to realize that we have done a lot for them, here on these postcards, they show their tenderness in return.”	Respect
Learning new skills made participants feel young	“We felt connected when this photo exhibition took place. We felt young. Not like older people. We were serving the guests like a 16‐year‐old teen.”	Fun

*Note*: *pseudonym.

**FIGURE 2:**
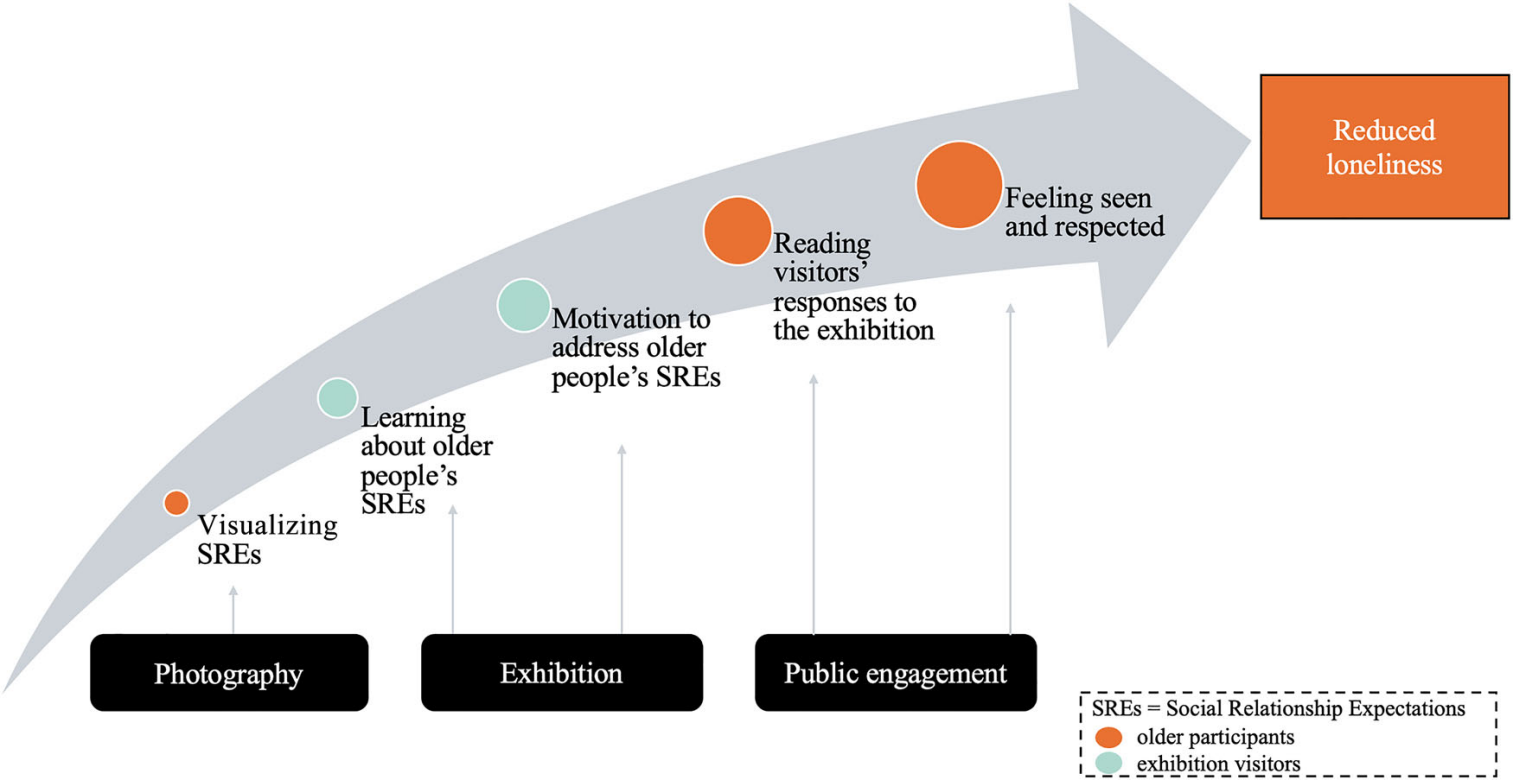
Intervention pathways to reduce loneliness. The figure describes how the three different intervention components (photography, the exhibition, and the public engagement [answers on postcards]) contributed to fulfilling social relationship expectations and reduced loneliness. The orange points describe outcomes experienced by participants of *amanane*; the green points describe outcomes experienced by visitors who attended the exhibition. The growing sizes of the dots indicate the growing importance of the elements for addressing generativity and respect, and for reducing loneliness. SREs, social relationship expectations.

**FIGURE 3:**
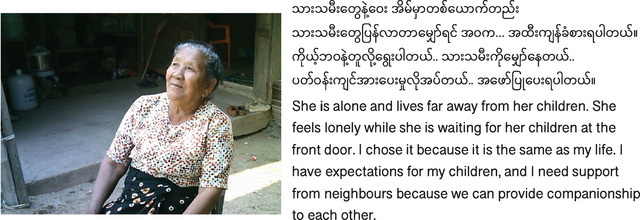
Example photo from the exhibition. The photo and caption that was shown at the exhibition illustrates older adults’ social relationship expectations (for children and neighbors) and their relationship to contextual factors, such as migration. The caption also shows that participants discovered similarities through photography (e.g., feeling lonely due to separation from children). © Daw Khin Zar Lwin (self‐chosen pseudonym).

In the following, we describe how the different components of *amanane* (photography workshops, exhibition, and public engagement) may have elicited change among participants and visitors, resulting in reduced loneliness among older migrants from Myanmar. Figure 2 illustrates one of the potential pathways through which participants felt less lonely after *amanane*. Participants learned to express their SREs (e.g., to be respected) through the photos and captions in the workshops and presented them to the public in the exhibition (Figure 3). Consequently, the public engagement component showed that the visitors learned about older adults’ expectations and were motivated to address these (e.g., by paying more attention and respect to older people, see section “Community outcomes”). Reading the postcards made older people feel understood, seen, valued, and ultimately, less lonely (Table 1).

I love the ones written by Thai people. I really appreciate when other nations show their compassion to our people.

Participants experienced reduced loneliness due to a sense of closeness between participants that was elicited by opening up to one another and sharing problems with each other, without feeling embarrassed. The group discussions in the workshops helped to get to know each other, while identifying similarities (e.g., importance of nature, family, and spirituality). The workshops unified people who previously thought they could not communicate due to not feeling comfortable speaking Burmese or because they were from different ethnic groups (Burmese and Karen). Having a sense of unity came up several times in the focus group discussion and was also exemplified in the photos taken (e.g., Figure 3). Importantly, the role of photography added a unique component to *amanane* for reducing loneliness. First, using the camera was a new experience for everyone. Although most participants had smartphones, none had used a digital camera before (including co‐facilitator B.L.). Acquiring new skills helped people feel young and proud. As some participants learned how to use the camera faster than others, people helped each other with taking photos during and in between the workshops. This open communication about how to use the camera also transferred to talking about everyday problems. In other words, photography helped participants open up about their personal lives, sharing joyful events, worries, and hardships. Photography also facilitated intergenerational contact, as older adults asked their grandchildren or other children from the neighborhood for support with taking photos (or were asked to teach them how to use the camera). Moreover, whereas group discussions were limited to the weekly workshops, participants were able to relive their memories at home by looking at the photos they took during the sessions. When asking participants whether it would be necessary to have cameras for an intervention to reduce loneliness in the future, they responded with conviction:

It [using cameras] is necessary if we start anew. Only then, they will, like me, get excited and united. We would get closer communicating on how to shoot photos, asking each other what is what and how.(…) But without a camera, it's hard to get friendly.

It [alleviating loneliness] is associated with a camera. It's a great feeling to go through the photos I've taken when I got back home. (…) At home, when feeling blue and lonely, we can just look at the photos taken again.

The decision to have an exhibition with photos and messages was another important component that reduced older adults’ loneliness. At first, some participants expressed a sense of insecurity about communicating their photographs, but quickly warmed up and eagerly engaged with others. Several participants expressed a change in personality following the exhibition, attributing their reduced loneliness to a change in mindsets (e.g., “learning to forgive and forget”). For example, U Tin Shwe's change of behavior at the exhibition was described as a surprise by his wife:

He didn't look tired serving others. He was giving a non‐stop presentation to every attendee. He never used to be like that. He is a man of few words. I hope there will be more exhibitions.

The exhibition was not only a learning opportunity for participants, where they learned to present their photos to a public audience; it also provided an opportunity to realizing generativity by meaningfully contributing to society. For instance, at the exhibition, participants told the first author that they felt happy because their participation in the project contributed to finishing her PhD and having a better future due to a higher educational degree. Generativity was also realized by sharing wisdom (e.g., how to use plants for medicinal purposes) and messages (e.g., to respect and listen to older people) directed at a younger audience (Table 2).

I gradually learnt from my work; how to do it, how to say it and so on. (…) There is no point to be nervous for the photo exhibition. Us older people took pictures and young people came to see it. It is great that they can learn from us.

### Community outcomes

Visitors who came to the exhibition provided answers on the postcards and video material that was used to evaluate community impact and public engagement. Among the visitors were Myanmar community members (family members and neighbors), Thai public health officers, and Thai and Myanmar university staff and students. Three main categories were identified from the responses on the postcards and video recordings: positive response to the exhibition, improved understanding of older people, and motivation to improve older people's lives.

#### Positive response to the exhibition

Myanmar visitors expressed positive emotions in response to the exhibition, including feeling happy, feeling grateful to be part of the experience, and feeling touched by the photos. Several visitors pointed out how they felt especially happy for older people to be able to participate in this project. As the format of a photo exhibition was new to all visitors, the experience was received with excitement.

I am very pleased that this photo exhibition gave older people happiness that I myself could not.

(Myanmar visitor at the exhibition)

Photography can convey so many meanings to others and make people understand each other more. It does not require a lot of questioning, but people can understand the meaning of the picture described. (…) I never thought about this perspective.

(Thai public health officer)

#### Improved understanding of older people

The exhibition helped both Thai and Myanmar visitors to better understand older people's daily lives as migrants, their values, desires, and SREs, especially for their children and grandchildren. Visitors learned about the importance of religion, nature, care, and family ties for older people's well‐being (i.e., learned how to make older people happy). Moreover, several visitors also gained a deeper understanding about the importance of psychological well‐being for general health.

There is love in a family, love in their culture, and love in the way of life. (…) The care for each other and generosity are still being given to anyone, no matter where they are. And there is the transfer of their experience, their knowledge, that continues to be given to their children, grandchildren, and people nearby. It makes us pay more attention to this aspect, regarding the dimension of health: It is not just about sickness, but we also need to look at the environment, the wellbeing, deep feelings, or foundation of life. These have the same effect on health.

(Thai public health officer)

#### Motivation to improve older people's lives

Visitors of the exhibition expressed a desire to take action in order to improve older people's well‐being. These actions included reducing loneliness by providing emotional support (e.g., “taking care of their mind”) and structural support (e.g., “provide suitable housing”), implementing activities for older adults while their children and grandchildren are at work (e.g., “take older people out to experience new things”), and respecting them.

As Thai people, we should respect and help those who are away from home as well as develop an approach to more interconnection and encouragement. (…) We should pay attention to taking care of older people, not let them be lonely. Older people should have a group of friends to talk to which will help with loneliness and take care of the surrounding environment.

(Thai visitor at the exhibition)

After visiting this exhibition, I pledge that in the future I will support older people emotionally.

(Myanmar visitor at the exhibition)

### Implementation challenges

Despite the positive outcomes, there were a few issues that had to be addressed while implementing the project, some of which entail contextual factors from the SRE framework (e.g., health, conflict).

#### Security concerns

In the initial information session before the first workshop, participants raised concerns about security issues and were willing to participate on the condition that there would not be any political discussions throughout the project. Following this initial session, participants experienced the research process as acceptable and felt safe and supported throughout the project. If this photovoice project were conducted in Yangon, the biggest city in Myanmar, participants agreed that it would be useful for participants to alleviate loneliness and feel united; however, there would be too many obstacles to successfully implementing the project. Participants anticipated less support from children and neighbors in a big city, as people would be busier with their jobs and not as familiar with each other. Regarding the current political situation following the military coup of 2021, it might also be dangerous for people to walk around with cameras and take photos, as it raises suspicion and ethical issues of what to photograph (e.g., “impossible; you will get questioned why you are taking photos. Not a good time.”).

#### Technical issues, time, and compensation

The cameras were experienced as user‐friendly and more convenient than smartphones, as one could easily print copies and have a large storage to take many photos and look at them. Unfortunately, the point‐and‐shoot cameras and memory cards were of low quality due to the limited budget, and several had to be replaced. These technical issues were stressful for participants, especially as they had never used a camera before. As such, weekly meetings with all participants were important in order to replace the cameras. Participants agreed that the timing of weekly workshops was ideal for older adults, compared to more (or less) often. One of the reasons for not having biweekly or monthly workshops was the worsening memory of older adults (e.g., “A weekly program can facilitate our memory recall, compared to a monthly program.”).

While some participants explained that they did not want to be compensated, as they saw the project as a learning opportunity, others referred to the money as benefit of taking part in the study, and either donated or kept it as a memory.

He keeps it as souvenir in his bag, the money, camera, and photos. Buddhists usually give away things in return if they receive help from donors. So, both can enjoy meritorious deeds despite being from different religions.

Despite the compensation being very moderate and the standard compensation fee suggested by the CHV, one participant felt pressured by receiving money to deliver good work.

When I came here, Sayarmalay [teacher] compensated me. I was worried about what would happen if I was not capable to do it well while being paid. I was growing a sense of anxiety seeing that I wouldn't be able to fulfill your expectations. I somehow felt irresponsible.

This participant was still working and had a relative who passed away recently, which distracted her from investing more time. Nevertheless, the facilitators perceived this participant as very actively engaged, both in the discussions, and the quality and number of photos taken. Because of the time constraints and technical issues of the cameras and memory cards, some participants were not able to take photos in the time between the workshops. Hence, having time to take photos during the workshops became an even more important component of the sessions (next to captioning and discussing the photos).

#### Vision and language barriers

After a few workshops, it became clear that some participants had vision problems. Before the exhibition took place, one participant approached the facilitators (S.C.A.‐K., B.L.) and asked whether they could be taken to the optician to get glasses. Three participants were taken to Mae Sai and got glasses. For some, it was their first ever trip to the city, which was a 15‐min drive from their home. This experience, although not initially part of the intervention, was very memorable and an immediate improvement for their daily lives and participation in the exhibition and evaluation session (e.g., “What is my favorite memory? It is going to be these glasses.”).

Another obstacle for active participation in the discussion was that the workshops were conducted in Burmese. Before the intervention, everyone indicated they spoke Burmese; still, in the workshops, some participants felt more comfortable communicating in Karen. Fortunately, B.L. was able to translate Karen to Burmese, so participants could explain and caption their photos in Karen.

#### Continuation of project

Participants could keep the camera after project completion and were eager to shoot more photos in their leisure time, for example, at the pagoda on public holidays (e.g., “I'm gonna take it with me wherever I go.”).

Yet, without S.C.A.‐K. and B.L. as facilitators, they did not want to continue their weekly meetings and workshops. Much of this projects’ success was dependent on the relationship that was established between the participants and facilitators. According to some participants, having new facilitators would make them unwilling to continue, as they were unsure whether they could establish a similar relationship with the new facilitators (e.g., “Because we became close already. We have a kind of past deed of merit (*ye set*). I'm not sure we will have this kind of chemistry with a new one.”).

## DISCUSSION

The present study evaluated the feasibility and preliminary effectiveness of a participatory loneliness intervention called *amanane* using photovoice with older Myanmar migrants living in Mae Sai, northern Thailand. Preliminary results suggest that photovoice can be implemented and coproduced with older migrants and showed high acceptability and a perfect completion rate. The intervention has the potential to reduce loneliness after 5 weeks of active participation in workshops and a photo exhibition, as reflected in the qualitative evaluation. Although the preliminary effectiveness must be interpreted with caution (given the small sample size), results indicate a perceived reduction in loneliness following the intervention and support findings from previous studies that argue for potential therapeutic effects of photovoice.^17^, ^20^, ^21^

The qualitative evaluation provided insights into potential mechanisms of change in loneliness. First, the workshops gave people the opportunity to open up about their personal lives through photography, which facilitated meeting their SREs for intimacy and support. In many Southeast Asian cultures, people tend to not talk about their personal problems with their neighbors, family, or friends, as they worry about putting a burden on them or making them feel bad, which is described by the commonly used term *anade* in Burmese.^27^ *Anade* is, therefore, closely related to feeling lonely, as one usually endures negative feelings alone without sharing or communicating them.^27^ The intervention was called *amanane* as this term is used by people from Myanmar to tell someone they should not have *anade* (e.g., by being able to speak their mind) and describes how people felt after opening up to others: feeling more supported and understood but also overcoming initial differences (regarding ethnicity and language). Second, participants learned to use a camera and take photos to share their stories with a diverse audience, which made them feel young, generative, and appreciated. Being actively involved in the intervention and presenting the photographs in an exhibition may have contributed to a more positive self‐perception as an older person, counteract ageism, and fulfill the expectations for generativity and respect. In a recent generativity intervention, older women with positive expectations regarding aging reported reduced loneliness after sharing written advice with younger people over 6 weeks.^38^ Hence, future studies evaluating the effectiveness of photovoice with older adults could benefit from assessing expectations regarding aging to evaluate potential moderating effects between generativity and loneliness. Third, as opposed to other loneliness interventions that aim at either skill development or group interactions,^3^ the bottom‐up approach of photovoice—its focus on active participation, between‐session interaction, and its diverse teaching styles with clear learning mechanisms—integrated all five active ingredients of successful loneliness interventions recently identified in a systematic review.^10^

Still, implementing photovoice requires flexibility and tailoring the intervention to participants’ individual needs. Unlike interventions with a set protocol or online delivery, photovoice is based on a trusting personal relationship that needs to be established between the facilitators and participants. Language skills and an in‐depth understanding of the cultural and political context are key to the successful implementation of participatory interventions like photovoice, especially in a setting affected by conflict. Co‐facilitating the intervention with an older adult was inherent to the successful delivery and establishing trust with participants. Participants pointed out that they especially enjoyed the intergenerational experience of having an early career researcher co‐facilitate photovoice, as this made them feel like they contributed to the researchers’ career (i.e., addressing generativity). Future interventions could benefit from emphasizing the intergenerational component of photovoice by having younger facilitators for older participants and focus even more on the expectation for generativity (by addressing topics that affect future generations, such as climate change). Creating an SRE scale to assess the expectations prior to the intervention will also help tailor *amanane* to participants’ expectations even better in future projects. Given the importance of flexibly adjusting workshops to participants’ learning speed and needs as well as the quality of the facilitator role to establish a personal connection, the question of whether photovoice (and other participatory interventions) can be scaled up poses a major challenge for future work.

The intervention was grounded in the SRE framework, a new lifespan theory of loneliness.^14^ Throughout the project implementation, several contextual factors proposed to be related to loneliness in the SRE framework became evident and needed to be addressed, including conflict (e.g., security concerns), migration (e.g., language barriers), health (e.g., bad eyesight), and poverty (e.g., no money for buying glasses). Like other photovoice projects,^17^ *amanane* may be limited by not reaching people who feel most lonely (e.g., people with health problems or who cannot leave their homes). Addressing some of these health problems before implementing the intervention could help improve inclusivity (e.g., by buying glasses for participants who needed them before the start of the project). These findings call for a focus on contextual factors in loneliness interventions and echo scholars who propose that loneliness is a societal issue, not merely a personal one.^39^, ^40^

This study has several limitations. First, participants were screened for inclusion with a culturally adapted Burmese DJG loneliness short scale that has not been validated in Myanmar yet. Hence, future studies should assess loneliness with a validated scale and compare pre‐ and post‐assessment scores. A pre‐ and post‐assessment of other outcomes, including SREs, depression, subjective health, and self‐perceptions of aging, could help to further tailor the photovoice intervention to personal expectations and improve understanding of the mechanisms of change in loneliness. Next, despite the perfect completion rate in this small sample, some participants reported time constraints due to work and care obligations, which is a common obstacle of participatory projects implemented with carers.^30^ Moreover, some participants worried about whether their photographs were good enough, reflecting an “anxiety to please,” which has previously been reported in other photovoice studies with older adults.^16^ As participants used cameras for the first time and we did not include follow‐up evaluations, it is unclear to which extent the positive outcomes were due to a novelty factor. Finally, we did not follow up about the action element of photovoice (e.g., whether visitors at the exhibition implemented their propositions to respect and include older people more), which may also affect older people's long‐term feelings of loneliness in their community and would be an important avenue for future research projects.

## CONCLUSION

Preliminary results of photovoice as an intervention for older people's loneliness are promising. Although the success of participatory interventions is more dependent on the facilitators’ ability to establish a personal connection and be flexible, *amanane* integrates a range of active ingredients, addresses all six SREs, and can be tailored to individual needs. This study lays the groundwork for larger randomized control trials with follow‐up assessments to test the feasibility, effectiveness, mechanisms of change, and sustainability of photovoice as a loneliness intervention.

## AUTHOR CONTRIBUTIONS

**Samia C. Akhter‐Khan**: Conceptualization; methodology; formal analysis; investigation; writing—original draft; project administration; funding acquisition. **Chanyanut Wongfu**: Investigation; writing—review and editing; project administration. **Nang Myat Pont Aein**: Investigation; writing—review and editing. **Ben Lu**: Investigation. **Matthew Prina**: Conceptualization; methodology; writing—review and editing; supervision. **Sirinan Suwannaporn**: Methodology; investigation; writing—review and editing; project administration; supervision. **Rosie Mayston**: Conceptualization; methodology; investigation; writing—review and editing; supervision. **Khin Myo Wai**: Conceptualization; methodology; investigation; writing—original draft; writing—review and editing; supervision.

## COMPETING INTERESTS

The authors have no competing interests to declare.

## Supporting information



Supporting Information

Supporting Information

Supporting Information

## Data Availability

The data that support the findings of this study are available from the corresponding author (S.K.) upon reasonable request.

## Footnotes

a
*Amanane*, derived from the typical Burmese concept *anade*, which prevents people from opening up to one another because of the worry to be a burden to others; *amanane* means to not have *anade* and is usually used in a family context, indicating that people can speak freely and do not have to worry about burdening others, allowing space for emotional intimacy.
