# Supplementary material for: The feasibility of using photovoice as a loneliness intervention with older Myanmar migrants
Source: Ann N Y Acad Sci. 2025 Jan 28;1544(1):65–77. doi: 10.1111/nyas.15270 (PMC11829318; doi:10.1111/nyas.15270)
Supplement: Supplementary file 1 — Supporting Information [file NYAS-1544-65-s001.docx]

**Supporting Material**

Supporting Table S1. Burmese adaptation of the De Jong Gierveld Loneliness Short Scale.

|  | No  မဟုတ်ပါ | More or less  အနည်း နဲ့ အများ | Yes  ဟုတ်ပါသည် |
| --- | --- | --- | --- |
| I experience a general sense of emptiness.  *I lack a sense of meaning in life.*  ဘဝကအဓိပ္ပာယ်မဲ့တယ်လို့ခံစားရတယ် |  |  |  |
| There are plenty of people I can rely on when I have problems  ကိုယ့်မှာ ပြဿနာတွေနဲ့ကြုံ လာတဲ့အခါ အားကိုး လို့ရတဲ့လူတွေ အများကြီးရှိတယ် |  |  |  |
| There are many people I can trust completely  ကိုယ့်မှာ အပြည့်အဝယုံကြည် လို့ရတဲ့သူတွေအများကြီးရှိတယ်။ |  |  |  |
| I miss having people around  *I wish I had more people around me.*  ငါ့နားမှာ လူတွေပိုပြီး ရှိစေချင်တယ် |  |  |  |
| There are enough people I feel close to  ကိုယ့်နဲ့ ရင်းနှီးတယ်လို့ခံစားရတဲ့သူ အလုံအလောက် ရှိတယ်။ |  |  |  |
| I often feel rejected  ကိုယ့်ကို ငြင်းပယ်ခံရတယ်လို့ မကြာခဏခံစားရတယ်။ |  |  |  |

*Note.* Following the recommendations for cultural adaptations of scales by Beaton et al., (2000), the six-item scale was translated by two independent Burmese native speakers (one of who was unfamiliar with the research questions/context) and back-translated by two other Burmese speakers who were fluent in English. Translators and authors of this paper (S.A., R.M., K.M.W.) met to compare and discuss the translations and back-translations. The first item of the scale “I feel a general sense of emptiness” was changed to “I lack a sense of meaning in life” as this was considered a more understandable and often-used term in Burmese. The scale was then piloted with five older Myanmar migrants living in Mae Sai district, Chiang Rai, using cognitive telephone interviews (including think-aloud technique and prompts about the understanding of each item) (by N.A.). We chose a more nuanced three-category item score to evaluate the responses for the DJG loneliness scale (which correlates strongly with the dichotomized item scores) (De Jong Gierveld & van Tilburg, 1999). The response scale was scored as following: “No” = 0; “More or Less” = 1; “Yes” = 2, resulting in a maximum score of 12.

Supporting Table S2. Checklist for reporting loneliness interventions by Schönmakers et al. (2024)

| **Checklist for reporting loneliness interventions (Schönmakers et al., 2024)** | **Mentioned section in manuscript** |
| --- | --- |
| 1. Full description of the SIL intervention:  a. Components of the intervention (with sufficient information to permit replication);  b. Intervention material: if the SIL intervention is highly structured (e.g. social skills training), training materials should be made available in appendices, if not (e.g. befriending services) any information shared  with participants and intervention deliverers should be public;  c. Frequency and duration of the intervention (e.g. number of sessions, frequency of sessions, duration of sessions, intensity of intervention, flexibility of schedule);  d. Delivery setting of the intervention (e.g. group, individual or mixed, in person, online or phone-based, any necessary infrastructure);  e. Expertise of the intervention deliverer (e.g. psychologist, assistant, volunteer) and any training given;  f. Assignment of the intervention deliverers to the participants; | a. Intervention components  b. Supplementary Material  c. Intervention components  d. Intervention components  e. Co-production  f. Intervention components / Supplementary Material |
| 2. Expected primary outcome(s) of the intervention:  a. Categorisation of SIL intervention (see Figure 1);  b. Exact outcome(s) and operational definitions (e.g. social isolation, loneliness, social support, health). | a. Evaluation  b. Introduction / Evaluation |
| 3. Explication of the programme theory:  a. Intervention strategy (e.g. social skills training, psychological interventions, social support interventions);  b. Description of the mechanisms by which the intervention is proposed to achieve its expected outcomes;  c. Existing SIL theories that inform the programme theory (See: Berkman et al., 2000; McHugh Power et al., 2018; Seemann, 2022; Stein and Tuval-Mashiach, 2015). | a. Program theory  b. Program theory / Supplementary Material  c. Program theory / Introduction (SRE) |
| 4. Provision of hypotheses:.  a. The expected effect of the SIL intervention on the main outcome is described (size, direction, time  before the effect takes place, duration of the effect, etc.); | a. Supplementary Material (ToC) |
| 5. Fidelity measures (if taken): For instance, adherence, fidelity to initial programme theory; tailoring or personalising of the intervention; modifications of the intervention; steps taken to improve implementation fidelity (see Carroll et al., 2007). | Changes to implementation |
| Study design  6. Information on the sample characteristics:  a. Characteristics, i.e. number of potential participants approached, number of gatekeeper organisations, number screened, number eligible prior to enrolment, sample size, sample selection and recruitment, a priori sample size estimation, non-response, country of intervention, mean age, age range, gender distribution, characteristics of the community;  b. Flowchart of participant recruitment, e.g. that provided by CONSORT-SPI (Grant et al., 2018);  c. Extent of missingness and how it was responded to. | a. Recruitment  b. Supplementary Figure 1  c. NA |
| 7. Identification of study design and any modifications made after the study started:  a. Complete schedule of outcome assessment and rationale provided for choice of measures;  b. If relevant details of randomisation and allocation processes (sequence generation, blinding strategies, implementation);  c. Inferential analytic methods chosen to look for an effect;  d. Any additional analyses not previously specified;  e. Details of process evaluations if relevant. | NA |
| Results  8. The baseline SIL of the interventions’ participants (e.g. mean social isolation and/or loneliness and their  standard deviation);  9. If applicable the baseline of other outcomes (for Category II interventions, baseline of the primary  outcome);  10. Sample size in analyses  11. Report of the study outcomes  a. Effect size and precision of the intervention effect  b. Harms or adverse effects | 8. Changes in loneliness  9. NA  10. Supplementary material  11. Qualitative evaluation and Feasibility |
| Discussion  12. Interpretation of study results  a. Clear contextualising in existing SIL intervention literature, and implications for SIL theories. | 12. Discussion and conclusion |

Supporting Figure S1. Theory of Change

Help identify and recruit older migrants from Myanmar in Mae Sai with the Burmese DJG loneliness short scale (i)

Fulfilling the expectation for generativity and respect (iii)

Reduced prevalence of loneliness in Mae Sai

Awareness for older migrants’ contributions on the policy level

Reduced loneliness among participants (iv)

Older Myanmar migrants feeling valued for their unpaid contributions (iii)

Increased community/global knowledge about older migrants’ contributions (v)

International photo exhibition

Local photo exhibition

Presentation of older people’s experiences as migrants (iii)

Fulfilling social relationship expectations support and fun (iii)

Visualisation of care contributed by older adults (iii)

IMPACT

LONG TERM OUTCOME

INTERMEDIATE OUTCOME

INTERVENTION

IDENTIFICATION

Community Health Volunteers (CHV) in post

Ongoing management and supervision

PhD supervisors (UK, Myanmar, Thailand)

Five photovoice workshops with older lonely migrants from Myanmar (ii)

Intervention coordinator (SA) & co-facilitator in post

RESOURCES

b

a

d

c

C

A

e

D

E

B

| **Assumptions**   1. A co-facilitator understands the situation of older migrants from Myanmar, has time, and is motivated to be trained in photovoice and co-conduct the workshop together with the intervention coordinator (SA). 2. CHV are engaged with the program and have time to recruit participants. 3. Enough older people feel lonely, have time, and are motivated to participate in weekly workshops over 5-6 weeks. 4. Older people want to share their photos and narratives publicly as a photo exhibition. 5. Policymakers, community members, and researchers attend the exhibitions and show interest in the project. | **Indicators**   1. Successfully recruit 10 participants who are over 50 years old, speak Burmese, and report loneliness on the culturally adapted Burmese version of the De Jong Gierveld Short Loneliness Scale (score >2). 2. Repeat five weekly workshops between January and March 2023 with one photovoice group (n=10). 3. Participants report fulfilled expectations and feeling valued in the evaluation post intervention (focus group). 4. People will feel less lonely following the exhibition (subjective evaluation). 5. Improved knowledge and awareness about older migrants from Myanmar and their contributions at the exhibition through post-cards with evaluative questions / open feedback. The public will perceive older Myanmar migrants as valuable contributors and understand that unpaid work is valuable and needs to be appreciated. |
| --- | --- |
| **Rationale**   1. Photovoice is a community-based participatory research method that combines photography and storytelling to share a person’s perspective and empower a society’s most vulnerable population (Wang & Burris, 1997), in this case, older migrants from Myanmar. Photovoice has been successfully implemented with older people in several studies (Mysyuk & Huisman, 2020). 2. According to the Social Relationship Expectations Framework (SRE) (Akhter-Khan et al., 2023), loneliness results from unmet expectations regarding social relationships: Proximity, Support, Intimacy, Fun, Generativity, and Respect. By engaging in an activity with other people from the same community who share similar experiences (migration, loneliness) and interests (photography, community activities), the expectation for proximity and fun should be fulfilled. 3. Engaging people in a group activity over several weeks can provide the opportunity for new friendships or intensifying existing ones. By sharing personal stories and lived experiences with peers, the expectations for support and intimacy can be addressed. 4. By showcasing the photographs, sharing their feelings and personal stories, being listened to, visualizing their unpaid productive activities, and perhaps evoking change in the community, the expectations for generativity (a meaningful contribution) as well as respect (feeling valued and respected for their contributions) may be fulfilled. 5. Photovoice has shown to decrease symptoms of depression in a study with women farmers in Nepal (MacFarlane et al., 2015). Contributing to society in forms of care and volunteering have been shown to be associated with reduced loneliness across several studies (Akhter-Khan et al., 2022). Meeting the six SRE (intermediate outcomes, see b) is hypothesized to lead to reduced loneliness (Akhter-Khan et al., 2023). 6. Visualising care is thought as one of the first steps towards valuing care in the field of feminist economics (Elson, 2017; Folbre, 2005). Photovoice has been implemented in Tanzania to visualise and value care provided by women (Chang et al., 2019). | |

Supplementary Figure 2. CONSORT flow chart of participant recruitment

**
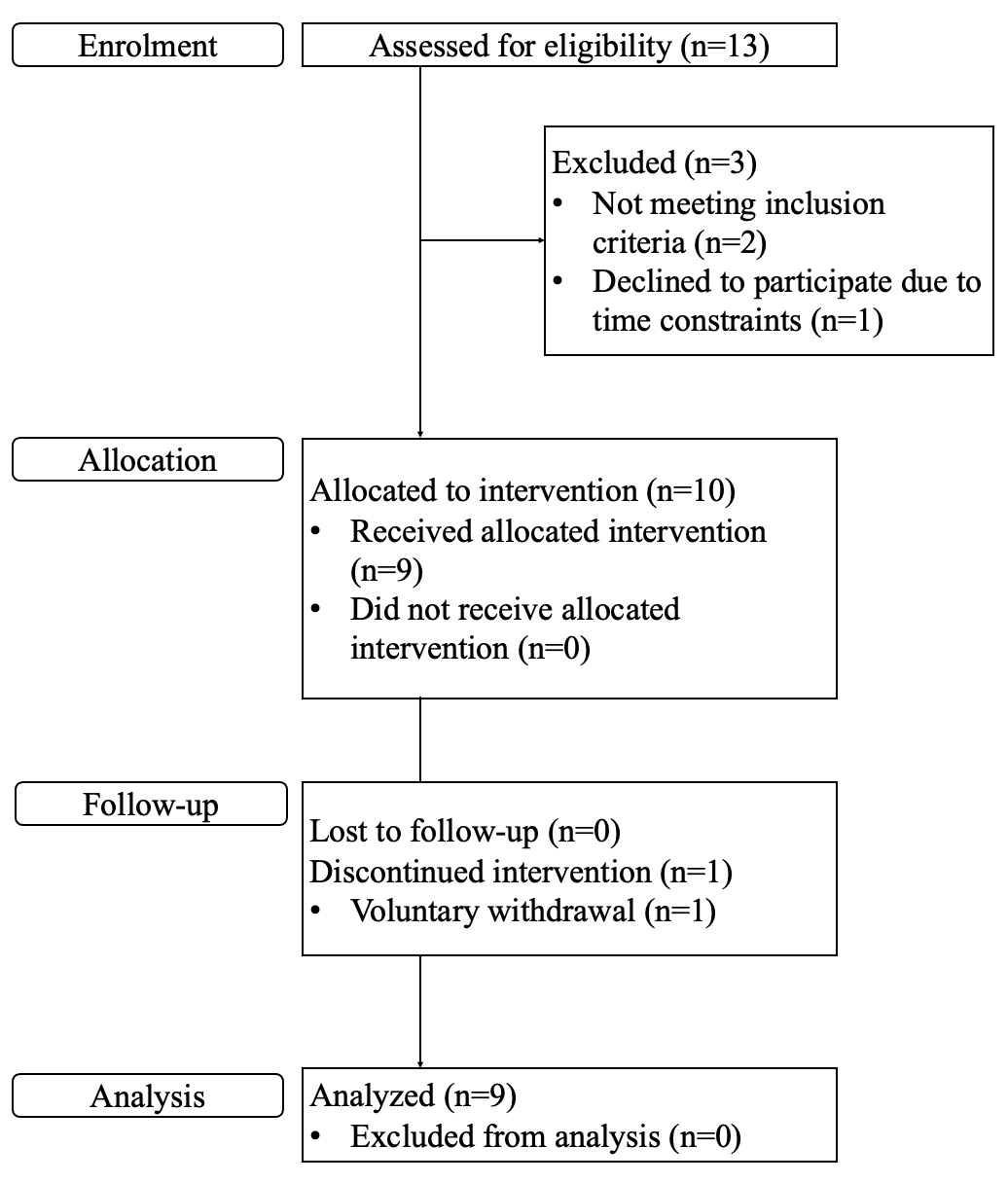
**

**References**

Beaton, D. E., Bombardier, C., Guillemin, F., & Ferraz, M. B. (2000). Guidelines for the Process of Cross-Cultural Adaptation of Self-Report Measures: *Spine*, *25*(24), 3186–3191. https://doi.org/10.1097/00007632-200012150-00014

De Jong Gierveld, J., & Van Tilburg, T. G. (1999). *Manual of the Loneliness Scale*. Methoden en technieken. https://doi.org/10.17605/osf.io/u6gck
