# Supplementary material for: The feasibility of using photovoice as a loneliness intervention with older Myanmar migrants
Source: Ann N Y Acad Sci. 2025 Jan 28;1544(1):65–77. doi: 10.1111/nyas.15270 (PMC11829318; doi:10.1111/nyas.15270)
Supplement: Supplementary file 2 — Supporting Information [file NYAS-1544-65-s002.docx]

**Interview Topic Guide**

1. Choose a pseudonym.

အမည်ဝှက်တစ်ခုရွေးပေးပါနော်။

1. What would be your main message for the exhibition that you would like to convey? Think about the photos you took for this week’s homework about things you want to change.

ပြပွဲမှာ မိမိအဓိကပေးချင်တဲ့ သတင်းစကားက ဘာဖြစ်မလဲ။ ဒီတစ်ပတ်အိမ်စာအတွက် ရိုက်လာတဲ့ဓာတ်ပုံတွေနဲ့ပတ်သက်ပြီး ပြန်စဥ်းစားကြည့်ပါ။ အဲ့ဓာတ်ပုံထဲမှာ ဘာတွေပြောင်းလဲချင်တာရှိပါသလဲ။

1. Present three photos asking the following questions for each photo:

ပုံ ၃ ပုံ ကိုတင်ပြ၍ ၁ ပုံချင်းစီအတွက် အောက်ပါမေးခွန်းများကို ဖြေပေးပါ။

- Why do you want to share this photograph?
- ဘာကြောင့် ဒီဓာတ်ပုံကို မျှဝေချင်တာလဲ
- Why is it important for you?
- ဘာကြောင့် ဒီပုံကို မိမိအတွက် အရေးကြီးတာလဲ
- What do you See here?
- ဒီပုံမှာ မိမိအနေနဲ့ ဘာမြင်တွေ့ပါလဲ
- What's really Happening here?
- ဒီပုံမှာ ဘာတွေဖြစ်ပျက်နေပါလဲ
- How does this relate to Our lives?
- ဒီပုံသည် မိမိတို့ဘဝနဲ့ ဘယ်လိုဆက်စပ်နေပါလဲ
- Why does this problem, concern, or strength Exist?
- ဒီပြဿနာက (သို့မဟုတ်) ဒီကောင်းတဲ့အရာက ဘာကြောင့်ရှိနေရတာလဲ။
- What can we Do about it?
- ဒါနဲ့ပတ်သက်ပြီး မိမိတို့ လုပ်နိုင်တာ ဘာရှိလဲ။
- Then, think of how to caption these (e.g., speak freely, associative words, poem, story). Think about the methods we learned last week.
- ပြီးတော့ ဒါတွေကိုခေါင်းစဥ်ဘယ်လိုတပ်လို့ရမလဲ စဥ်းစားပေးပါ (ဥပမာ-လွတ်လပ်စွာပြောဆိုပါ၊ ဆက်စပ်စကားလုံးများ၊ ကဗျာ၊ ဇာတ်ကြောင်း)။ ပြီးခဲ့တဲ့အပတ်တုန်းက ကျမတို့ လေ့လာသိရှိ ခဲ့တဲ့နည်းလမ်းတွေကို ပြန်စဥ်းစားကြည့် ပေးပါရှင်။

1. General questions for all photographs presented:

တင်ပြထားတဲ့ ပုံတွေအားလုံးအတွက် အထွေထွေမေးခွန်းများ

- What do these photographs represent in terms of positive and negative aspects which support or do not support you to feel valued as an older person in your community?
- မိမိရပ်ရွာမှာရှိတဲ့ အသက်ကြီးပိုင်းအဘိုးအဘွားတွေ တန်ဖိုးထားခံရစေရန်အတွက် အကူအညီဖြစ်စေမယ့် အပြုသဘောဆောင်တဲ့အရာတွေနဲ့ပတ်သက်ပြီး ဒီပုံတွေက ဘာတွေကိုတင်ပြထားလဲ။ (သို့မဟုတ်) အကူအညီမဖြစ်စေမယ့် အဖျက်သဘာဆောင်တဲ့ အရာနဲ့ပတ်သက်ပြီးရော ဘာတွေတင်ပြထားသလဲ။
- How do you think that these photographs/aspects you portrayed may be help to supporting other older people to age healthily and feel valued as an older person in your community?
- မိမိတို့ရိုက်လာတဲ့ ပုံတွေ/ရှုထောင့်တွေက ရပ်ရွာရှိ အခြားအဘိုးအဘွားတွေကျန်းမာ စွာနေထိုင်ဖို့ရန် နှင့် တန်ဖိုးထားခံရစေရန်အတွက် ဘယ်လိုတွေ အထောက်အပံ့ဖြစ်နိုင် တယ်လို့ ထင်ပါသလဲ။
- Imagine that there was the possibility to make your neighbourhood/local community a better place for older people to feel valued and respected by their community. In this, imagine that you have all the resources to make it possible. What would you do?
- မိမိတို့ရဲ့ လက်ရှိနေထိုင်ရာ ပတ်ဝန်းကျင်/ရပ်ရွာကို သက်ကြီးရွယ်များအား လေးစားတန်ဖိုးထားသော ပို၍ကောင်းမွန်သော ဝန်းကျင်တစ်ခုအဖြစ် ဖန်တီးပေးဖို့ ဖြစ်နိုင်တဲ့အလားအလာရှိတယ်လို့ စိတ်ကူးယဥ်ကြည့်ပါ။ အဲ့လို ဖန်တီးပေးရန်အတွက် လိုအပ်သည့်ရင်းမြစ်များအားလုံး မိမိတို့မှာရှိတယ်လို့ ထပ်မံစိတ်ကူးယဥ်ကြည့်ပါ။ အဲ့လိုအနေအထားမှာ မိမိတို့အနေဖြင့် ဘာတွေလုပ်ပေးပြီး ဖန်တီးမလဲ။

1. Are there any photographs that you might have wanted to take but you did not? If yes, can you tell me more about that?

မိမိအနေဖြင့် ရိုက်ချင်ခဲ့သော်လည်း မရိုက်ခဲ့ရသောပုံများရှိပါသလား။ ရှိတယ်ဆို အဲ့ဒါနဲ့ပတ်သက်ပြီး ကျမကို ပြောပြပေးနိုင်မလား.

1. Consent needed? Bring printed consent forms and let others sign by Sunday.

သဘောတူညီချက်လိုပါသလား။ ပရင့်ထုတ်ထားတဲ့ သဘောတူညီချက်ဖောင်ကိုယူခဲ့ပါ။ လာမယ့် တနဂ်နွေနေ့လောက်မှာ တခြားသူတွေကို လက်မှတ်ပေးထိုးရပါမယ်။

1. For an exhibition in London at King’s College, would you like to say something in a video about your experience participating in this project? (You can also turn around, so we do not see your face if you do not want to be identified.)

အဘိုးအဘွားတို့ ဒီစီမံကိန်းမှာပါဝင်ခဲ့တဲ့ အတွေ့အကြုံတွေနဲ့ပတ်သက်ပြီး လန်ဒန် ကင်းကောလိပ်မှာ ကျင်းပမယ့်ပြပွဲအတွက် ဗွီဒီယိုထဲမှာ တစ်ခုခုပြောချင်ပါသလား။ (မိမိတို့ကို ဘယ်သူဘယ်ဝါဆိုတာမသိစေချင်ရင် ဟိုဘက်လှည့်ပြီးပြောလို့ရပါတယ်၊ မျက်နှာမမြင်ရတော့ဘူးပေ့ါနော်)

- What did you learn during this project? How did the project benefit you or others in your community? How will this project change your future life or perspective on life?
- ဒီစီမံကိန်းမှာပါဝင်ခဲ့တဲ့ တလျှောက်ဘာတွေ လေ့လာသိရှိခဲ့ရလဲ။ ဒီစီမံကိန်းကနေ မိမိအတွက် (သို့) မိမိရပ်ရွာရှိတခြားသူတွေအတွက် ဘယ်လိုအကျိုး ကျေးဇူးတွေရှိ ခဲ့ပါသလဲ။ ဒီစီမံကိန်းက မိမိရဲ့ အနာဂတ် (သို့) ဘဝအပေါ်မြင်တဲ့ မိမိတို့ရဲ့ အမြင် စတာတွေကို ဘယ်လို ပြောင်းလဲစေလိမ့်မလဲ။
