# Supplementary material for: The feasibility of using photovoice as a loneliness intervention with older Myanmar migrants
Source: Ann N Y Acad Sci. 2025 Jan 28;1544(1):65–77. doi: 10.1111/nyas.15270 (PMC11829318; doi:10.1111/nyas.15270)
Supplement: Supplementary file 3 — Supporting Information [file NYAS-1544-65-s003.docx]

­­ **Workshop Plan**

**အလုပ်ရုံဆွေးနွေးပွဲ အစီအစဥ်**

**Day 1 ပထမနေ့**

***Materials:*** *Snacks, cameras with SD cards, printed photos, activity log, consent forms, information sheet, coffee plants, 200 Bath per participant, homework activity printed out*

*ပစ္စည်းပစ္စယများ- မုန့်၊ အက်စ်ဒီကဒ်နဲ့ ကင်မရာများ၊ ပရင့်ထုတ်ထားသည့်ပုံများ၊ လုပ်ဆောင် ချက် မှတ်တမ်း၊ သဘောတူညီချက်ပုံစံ၊ သတင်းအချက်အလက်စာရွက်၊ ကော်ဖီပင်များ၊ တစ်ယောက် ဘတ် ၂၀၀၊ ပရင့်ထုတ်ထားသည့်အိမ်စာ*

1. Introduction Samia and Ben Lu

ဆန်မီရာနှင့် အဘွားအင်ဒူတို့ မိတ်ဆက်ခြင်း အစီအစဥ်ခြင်း

- Who we are

ကျွန်မတို့က ဘယ်သူတွေလဲ

- Thank you for coming

ခုလိုကြွရောက်ပေးတဲ့အတွက် ကျေးဇူးတင်ပါတယ်

- Project will be about learning photography and being able to tell your own story through photos, the aim is not to give work (but 200 Bath per session)
- အခု စီမံကိန်းမှာ ဓာတ်ပုံဘယ်လိုရိုက်ရမလဲဆိုတာလေ့လာရမယ်၊ ဓာတ်ပုံတွေကနေ တဆင့် အဘိုးတို့အဘွားတိုရဲ့ အကြောင်းတွေကို ဘယ်လိုတွေပြောနိုင်လဲဆိုတာကို လေ့လာရမယ်။ ကျွန်မတို့ရဲ့ စီမံကိန်းကနေ အဘိုးတို့အဘွားတို့ကို မည်သည့်ဝင်ငွေရ အလုပ်မှပေး မှာ မဟုတ်ပါဘူး။ ဒါပေမယ့် အခုလိုအချိန်ပေးပြီးပါဝင်ပေးတဲ့အတွက် အဘိုးအဘွားတို့ကို လေးစားအသိအမှတ်အပြုတဲ့အနေနဲ့ အခုဆွေးနွေးပွဲပြီးသွားရင် တစ်ယောက်ကို ဘတ်၂၀၀စီ ဂါရဝပြုသွားမှာဖြစ်ပါတယ်။
- In the future, Samia will write about this work for her thesis
- နောင်တချိန်ကျရင် ယခုစီမံကိန်းကနေရလာတဲ့ အချက်အလက်တွေကိုတော့ ကျမဘွဲ့အတွက် လိုအပ်တဲ့ကျမ်းစာတစ်စောင်ရေးမှာဖြစ်ပါတယ်။
- This is a safe space, we don’t talk about what people share here to others outside of the group so that everyone feels comfortable
- ဒီနေရာက လုံခြုံတဲ့နေရာတစ်ခုပါ၊ ဒီမှာ အဘိုးအဘွားတို့ ပြောတဲ့အကြောင်းတွေကို အပြင်ကလူတွေကို ပြန်ပြောပြမှာမဟုတ်ပါဘူး၊ ဒါကြောင့် သက်သောင့်သက်သာနဲ့ ပြောဆိုဆွေးနွေးလို့ရပါတယ်။

1. Consent forms (သဘာတူညီချက် ဖောင်ပုံစံ)

- Ben Lu reads out every item of the consent forms, makes sure they are understood and agreed upon, before participants tick and sign
- အဘွားအန်ဒူကနေ သဘောတူညီချက် ဖောင်ပုံစံထဲမှာပါတဲ့ တစ်ချက်ခြင်းစီကို ဖတ်ပြမှာဖြစ်ပါတယ်။ ပြီးရင် ဒီက အဘိုးအဘွားတို့က အမှန်လေးတွေခြစ်မယ်၊ လက်မှတ်လေးတွေထိုးပေးရမှာဖြစ်ပါတယ်၊
- Any questions? မေးစရာများရှိပါသလား
- Start recording session မှတ်တမ်းတင်ခြင်း စတင်ပါတော့မယ်

1. Ice breaker with introductions ပျော်ရွှင်စရာမိတ်ဆက်အစီအစဥ်

- Name, age, family members, how many years in Thailand, experience with photography, motivation to participate
- နာမည်၊ အသက်၊ မိသားစုဝင်များ၊ ထိုင်းမှာနေတာဘယ်နှယ်နှစ်ရှိပြီလဲ၊ ဓာတ်ပုံရိုက်တဲ့အတွေ့အကြုံ၊ ဒီအစီအစဥ်မှာပါဝင်ဖြစ်ခဲ့တဲ့အကြောင်း

1. Visual literacy: provide photos to choose from ရွေးချယ်ဖို့ ဓာတ်ပုံတွေပေးမယ်

- What do you see? How does it make you feel? ဓာတ်ပုံထဲမှာ ဘာမြင်လဲ။ ဒီဓာတ်ပုံကိုကြည့်ပြီး ဘယ်လိုခံစားရလဲ။
- Circulate same photo and let every participant add a new word to describe it
- ပုံတစ်ပုံတည်းကိုပဲ တစ်ယောက်ပြီးတစ်ယောက်ကိုပြမယ်၊ ပြီးရင် ဒီဓာတ်ပုံက ဘာကိုဖော်ပြနေတာလဲဆိုတာကို တစ်ယောက်နဲ့တစ်ယောက် မတူတဲ့စကားလေးတစ်လုံးစီပြောပြပေးပါ။

*Tea Break (နားချိန်)*

1. Taking photos introduction: ဓာတ်ပုံရိုက်ကူးရန် သင်ကြာပေးခြင်း

- On/Off button, shutter button အဖွင့်/အပတ်ခလုတ်၊ ရှပ်တာခလုတ်
- Framing (what to include and exclude) ဘာတော့ထည့်ရိုက်မယ်၊ဘာတော့ထည့်မရိုက်ဘူး
- Focus (decide where you want the focus to be, front or back) ကိုယ်ရိုက်မယ့်နေရာကို ချိန်ပါ၊ အနောက်လား သို့မဟုတ် အရှေ့လား စသဖြင့် ချိန်ပါ
- Follow through (press shutter button and don’t move for a few seconds) (ရှပ်တာခလုတ်ကို ၂စက္ကန့်လောက် နှိပ်ထားပါ၊ ကင်မရာကို မရွေ့ပါနဲ့ ငြိမ်အောင်ထားပါ)
- Flash/light (where is the light coming from? Don’t take photos against the light). ဘယ်ကနေအလင်းရောင်လာတာလဲကြည့်ပါ၊ အလင်းရောင်ဘက်ကို ကင်မရာ ချိန်ပြီး မရိုက်ပါနဲ့)

1. Ethical considerations စောင့်ထိန်းရမယ့်ကျင့်ဝတ်များ

- Can you take photos anywhere of anyone at any time or could there be problems?
- အချိန်မရွေး နေရာမရွေး ဘယ်သူကိုပဲဖြစ်ဖြစ် ရိုက်လို့ရနိုင်လား၊ ဒါမှမဟုတ် အဲ့လိုလုပ်ရပ် ပြဿနာတက်နိုင်လား။
- When taking photos at work or of other people ask for permission first (also in this group).
- အလုပ်ခွင်မှာပဲဖြစ်ဖြစ် သို့မဟုတ် တစ်ခြားသူတွေကိုပဲဖြစ်ဖြစ် ဓာတ်ပုံရိုက်ရင် ခွင့်ပြုချက်အရင်တောင်းပဲ (အခုဒီအဖွဲ့ထဲမှာရိုက်ရင်လည်းပဲ ခွင့်တောင်းပါ)
- Example: photo of a person stealing, photo of a person crossing the border illegally -> consequences for everyone involved?
- ဥပမာ- ခိုးဝှက်နေတဲ့သူတစ်ယောက်ယောက်ရဲ့ ပုံကို ရိုက်ရင်၊ တရားမဝင်နယ်စပ်ဖြတ်ကျော်လာတဲ့သူတစ်ယောက်ယောက်ကို ဓာတ်ပုံရိုက်ရင် ဒီမှာ ပါနေတဲ့လူတွေအတွက် နောက်ဆက်ဆွဲဘာပြဿနာတွေရှိနိုင်လဲ
- Ground rule: before taking photos of people, ask them if it’s okay, and try to take photos where people are not identifiable
- မြေပြင်စည်းကမ်းများ-ဓာတ်ပုံမရိုက်ခင် ခွင့်ပြုချက်တောင်းပါ။ ပုံထဲကသူသည် ဘယ်သူဘယ်ဝါဖြစ်တယ်ဆိုတာကို ခန့်မှန်း၍မရအောင်ရိုက်ပါ။

1. Handing out cameras

- Activity log with signature
- ကင်မရာများအား နံပါတ်တပ်ခြင်း
- Try to take some photos and use the on/off button and shutter (walk around to help people)
- အဖွင့်/အပိတ်ခလုတ်နှင့် ရှပ်တာခလုတ်များအား အပိတ်/အဖွင့်လုပ်ကြည့်ပြီး ပုံအချို့ စမ်းရိုက်ပါ။
- Activity: take five photos of the coffee plant from different angles, lighting, closeness etc.
- ကော်ဖီပင်အား မတူညီသော ရှုထောင့်၊ အလင်းရောင်၊အနီးအဝေး တို့ဖြင့် ပုံ၅ပုံ ရိုက်ပေးပါ။ (ဆန်မီယာမှ လှည့်ပတ်ကြည့်ရှုပြီး အကူညီလိုသူကို ကူပေးမည်)

*Tea break (while Samia loads photos to computer) နားချိန်(ဆန်မီယာမှ ကွန်ပျူတာထဲကို ဓာတ်ပုံတွေထည့်နေမည်)*

- Look at some coffee plant photos and discuss how framing (distance/angles) can change the photograph
- ကော်ဖီပင်ပုံတွေကိုကြည့်မယ်၊ မတူညီသောရှုထောင့်များမှ ရိုက်ကူးထားသည့် ပုံသည် ဘယ်လိုပြောင်းလဲသွားသလဲဆိုတာကို ဆွေးနွေးမယ်။

1. Homework: Treasure Hunt အိမ်စာ

Take one photo of each category by the next session:

အောက်မှပြောမယ့် တစ်ချက်ချင်းစီအတွက် ပုံတစ်ပုံစီရိုက်ပေးပါ

1. Something that is your favorite color

မိမိကြိုက်သော အရောင်ပါသည့်တစ်ခုခု၏ ပုံ

1. A pattern

ဒီဇိုင်းလေးတွေပါနေတဲ့ ပုံ (ဥပမာ-အစင်းကြောင်းလေးတွေ၊ အဝိုင်းပုံလေးတွေ၊ ပန်းလေးတွေပါတဲ့ ဒီဇိုင်းပုံ)

1. A portrait

လူပုံ

1. Something natural

သဘာဝပုံ

1. A detail you think no one else will have noticed

မိမိပဲသတိထားမိပြီး တခြားသူတွေသတိမထားမိတဲ့ တစ်ခုခုရဲ့ပုံ (ဥပမာ- သစ်ကိုင်းလေးတွေမှာ ပုရွတ်ဆိတ်လေးတွေသွားနေတာကို ကိုယ်ပဲသတိထားမိပြီး တခြားတွေက သတိမထားမိတာမျိုး၊ အဲ့လိုပုံလေးတွေရိုက်မယ်)

- Any questions? (We should print this out as a task and give it to them)
- မေးစရာမေးခွန်းရှိပါသလား(ဒါကို ပရင့်ထုတ်ပြီး သူတို့ကိုပေးမယ်)

1. Give money to participants (200 each)

တစ်ယောက်ကို ဘတ် ၂၀၀ စီပေးမယ်

1. Remind participants of date and time for next workshop and call me if anything comes up (number on information sheet).

နောက်ထပ်အလုပ်ရုံဆွေးနွေးပွဲအတွက် ရက်နဲ့အချိန်ကို သတိပေးမယ်။ တစ်ခုခုဖြစ်ခဲ့လို့ရှိရင် ကျမကို ဖုန်းခေါ်လို့ရပါတယ်( ဖုန်းနံပါတ်က သတင်းအချက်အလက်စာရွက်မှာရှိပါတယ်)

**Day 2 (ဒုတိယနေ့)**

***Materials:*** *Snacks, ball, printed photos, 200 Bath per participant, homework activity printed out ပစ္စည်းပစ္စယများ-မုန့်၊ ဘောလုံး၊ ပရင့်ထုတ်လာတဲ့ဓာတ်ပုံ၊ တစ်ယောက်ကို ဘတ်၂၀၀၊ ပရင့်ထုတ်လာတဲ့ အိမ်စာ*

1. Ice breaker: Choose a photo that fits your mood today (from provided photo selection). အဘိုးအဘွားတို့ရဲ့ ဒီနေ့စိတ်ခံစားချက်နဲ့ကိုက်ညီမယ် ဓာတ်ပုံတစ်ပုံကို ရွေးပေးပါ( ရွေးချယ်ဖို့ပေးထားတဲ့ပုံတွေထဲမှ)

- What do you see on the photo? Why did you choose it?
- ဒီဓာတ်ပုံမှာဘာတွေ့လဲ၊ ဘာကြောင့်ဒီဓာတ်ပုံကိုရွေးရတာလဲ။
- How do you feel today? How was your week?
- ဒီနေ့မှာ ဘယ်လိုခံစားရလဲ။ ဒီတပတ်ထဲမှာရော ဘယ်လိုနေလဲ အဆင်ပြေရဲ့လား။
- Motivation to participate
- ဘာလို့ ဒီပွဲမှာပါဝင်ဖြစ်သွားတာလဲ။

1. Reflection on past week: How was the first experience as a photographer? ပြီးခဲ့တဲ့အပတ်က အတွေ့အကြုံများကို ပြန်လည်သုံးသပ်ခြင်း- ဓာတ်ပုံရိုက်တဲ့ ပထမဆုံးအတွေ့အကြုံက ဘယ်လိုနေလဲ။

- Were there any technical problems with using the camera?
- ကင်မရာသုံးတဲ့နေရာမှာရော နည်းပညာအခက်အခဲတွေရှိခဲ့လား
- Were there any situations where there were or could have been ethical issues?
- စောင့်ထိန်းရမည့်ကျင့်ဝတ်ပိုင်းဆိုင်ရာကိစ္စရပ်များနှင့်ပတ်သက်ရင်ရော ဘယ်လိုအခြေအနေရှိခဲ့လဲ။
- Did you ask other people for consent before taking a photo? How did that go?
- ဓာတ်ပုံမရိုက်ခင် ခွင့်ပြုချက်တွေယူခဲ့လား။ အဲ့လိုခွင့်ပြုချက်ယူတော့ အဆင်ပြေခဲ့ရဲ့လား ဘယ်လိုအခြေအနေရှိခဲ့လဲ။

1. Activity in pairs: Discuss with your partner what options there could be to take a portrait without being able to identify the person. Then, take a portrait of yourself / your partner without showing your/their face. *(After, Samia loads photos to computer)*

အတွဲလိုက် ဆောင်ရွက်ခြင်း- ဘယ်သူဘယ်ဝါဆိုတာကို ခွဲခြား၍မရတဲ့ ဓာတ်ပုံများကို ရိုက်ကူးနိုင်သည့်နည်းလမ်းများအား မိမိရဲ့ အဖော်နဲ့ ဆွေးနွေးပါ။ ပြီးတော့မိမိရဲ့ပုံ(သို့မဟုတ်) မိမိအဖော်ရဲ့ ပုံကို မျက်နှာမပြဘဲ ရိုက်ကြည့်ပါ။(ဒါတွေပြီးရင် ဆမ်မီယာက ပုံတွေကို ကွန်ပျူတာထဲထည့်မယ်)

- Discuss in group: What are ways you can take portraits without showing the face of a person? (e.g., think of objects that the person uses every day or likes, think of taking photos of different body parts, think of taking photos from behind).
- အုပ်စုဖွဲ့ဆွေးနွေးခြင်း- လူတစ်ယောက်ရဲ့ ပုံကို မျက်နှာမပြဘဲ ရိုက်လို့ရနိုင်တဲ့ နည်းလမ်း ဘာတွေရှိလဲ။ (ဥပမာ-လူတစ်ယောက် နေ့တိုင်းအသုံးပြုတဲ့ (သို့မဟုတ်) ကြိုက်နှစ်သက်တဲ့ အရာဝတ္ထုများ၊ မတူညီတဲ့ခန္ဓာကိုယ်အစိတ်အပိုင်းများ၊ နောက်ကနေရိုက်လို့ရနိုင်တဲ့ပုံများ စသဖြင့်)
- Present portraits to the group while letting the person explain their portrait and why they chose to take this photo to represent themselves
- အဖွဲ့တွေဆီ ပုံတွေကို ပြပါ။ ပြီးရင် သူတို့ရဲ့ပုံအကြောင်းနဲ့ ဘာကြောင့် ဒီပုံကိုရိုက်ဖို့ ရွေးချယ်ခဲ့ရသလဲဆိုတာကို ရှင်းပြခိုင်းပါ။

*Dinner (while Samia loads photos from Treasure Hunt to computer)*

*ညနေစာ (ဆမ်မီယာသည် ထရက်ရှာဟန့်မှ ဓာတ်ပုံများကို ကွန်ပျူတာထဲသို့ ထည့်နေမည်)*

1. Group activity “working with words”: Provide a word to the group (e.g., aging), throw a ball to someone, let every person say something associated with aging and give the ball to the next person.

“စကားလုံးများဖြင့် အလုပ်လုပ်ခြင်း” အုပ်စုဖွဲ့လုပ်ဆောင်ချက်- စကားလုံးတစ်လုံးကို အဖွဲ့ကို ပြောပြမယ်(ဥပမာ-အသက်အရွယ်ကြီးခြင်း)၊ ဘောလုံးတစ်လုံးကို တစ်ယောက်ထံကို ပစ်ပေးလိုက်မယ်၊ အဲ့လူက အသက်အရွယ်ကြီးခြင်းနဲ့ ဆက်စပ်နေတဲ့ တစ်ခုခုကိုပြောမယ်၊ ပြီးရင် သူကနေ နောက်တစ်ယောက်ကို ဘောလုံးကိုထပ်ပစ်မယ်။

- Realize that a word can have many meanings and be understood differently by everyone.
- စကားလုံးတစ်လုံးမှာ အဓိပ္ပာ​ယ်အများကြီးရှိပြီး တစ်ယောက်ကို တစ်မျိုးစီ နားလည်ကြတယ်ဆိုတာ သတိထားမိမယ်။
- One photo represents a thousand words: if one word can be understood in so many ways, imagine how many interpretations there are for one photo. That’s why it’s important to provide a context or story for a photo (i.e., describe a photo with captions).
- ဓာတ်ပုံတစ်ပုံသည် ထောင်ပေါင်းများစွာသောစကားလုံးများကို ဖော်ပြနေပါတယ်- စကားလုံးတစ်လုံးကို အမျိုးအမျိုးနားလည်ကြမယ်ဆိုရင် ဓာတ်ပုံတစ်ပုံကိုလည်း အဓိပ္ပာယ်အမျိုးမျိုးဖွင့်နိုင်တယ်ဆိုတာ တွေးကြည့်နိုင်ပါတယ်။ ဒါကြောင့် ဓာတ်ပုံတစ်ပုံကို ဇာတ်ကွက်ဇာတ်လမ်းညွှန်းတဲ့ စာတန်း ထည့်ဖို့ အရေးကြီးပါတယ်(ဆိုလိုချင်တာက ပုံကို ဇာတ်လမ်းခေါင်းစီးစာတန်းနဲ့အတူ တင်ပြပေးရမယ်)

1. Present Treasure Hunt photos: *ထရက်ရှာဟန့် ဓာတ်ပုံများ ပြခြင်း*

- Let every person choose one photo to present from their 5 categories (without saying which category it belongs to).
- (ဘယ်ပုံက ဘယ်အမျိုးအမည်ကို ဖော်ပြတယ်ဆိုတာကို မပြောစေဘဲ)

လူတိုင်းကို အမျိုးအမည် ၅ ခုမှ ပုံတစ်ပုံ ရွေးစေမယ်။

- Let other people guess which category the photo belongs to and why.
- ရွေးလိုက်တဲ့ပုံက ဘယ်အမျိုးအမည်မှာ ပါနေလဲ၊ ဘာကြောင့်လဲ ဆိုတာကို တခြားသူတွေကို ခန့်မှန်းစေမယ်။
- Then, let presenter explain which category, what they photographed, and why.
- အဲ့ဒီ့နောက် တင်ဆက်သူကို ပုံက ဘယ်အမျိုးအမည်ထဲပါတာလဲ၊ သူတို့ဘာအကြောင်းကို ရိုက်ခဲ့တာလဲနဲ့ ဘာကြောင့်ရိုက်ခဲ့လဲဆိုတာကို ရှင်းပြေစေမယ်။
- What do all photographs communicate (is there a common theme)?
- *ဓာတ်ပုံတွေအားလုံးက ဘာကိုပြောပြနေတာလဲ (အကြောင်းအရာတူတစ်ခုကို ဖော်ပြရဲ့လား)*
- Discuss experience of taking photos for this activity. Was it fun? Was it difficult?
- ယခုဆောင်ရွက်ချက်အတွက် ဓာတ်ပုံတွေရိုက်ကူးခဲ့တဲ့ အတွေ့အကြုံများကို ဆွေးနွေးပေးပါ။ ပျော်စရာကောင်းခဲ့လား။ ခက်ခဲခဲ့လား။

1. Homework: A day in my life.

အိမ်စာ-ဘဝထဲမှ နေ့တစ်နေ့

Take photos that represent a typical day in your life. You can take as many photos as you wish. Imagine you want to show someone outside of your village what your life looks like as an older person from Myanmar living in Thailand. You might want to show them what you do every day, who you spend time with, how you provide care to others or the environment.

ပုံမှန်နေ့လေးတစ်နေ့ကို ဖော်ပြပေးမယ့် ပုံတွေရိုက်ပေးပါ။ ပုံအရေအတွက်ကတော့ မိမိဆန္ဒရှိသလောက် ရိုက်နိုင်ပါတယ်။ ထိုင်းနိုင်ငံမှာ နေထိုင်နေတဲ့ မြန်မာပြည်သားအဘိုးအဘွားတစ်ဦးရဲ့ ဘဝသည် ဘယ်လိုပုံစံမျိုးလဲဆိုတာကို အပြင်လူတစ်ဦးဦးအားပြောပြနေသလိုမျိုး စိတ်ကူးပြီး ရိုက်ပါ။ မိမိရဲ့ နေ့စဥ်ဘဝမှာ ဘာတွေလုပ်လေ့ရှိသလဲ၊ ဘယ်သူတွေနဲ့ အချိန်ဖြုန်းလေ့ရှိသလဲ၊ အခြားသူများ (သို့)ပတ်ဝန်းကျင်အား မိမိအနေဖြင့်ဘယ်လိုတွေကူညီစောင့်ရှောက်ပေးနေလဲ ဆိုတာကို ပြောပြနေတာမျိုးပေါ့။

- Any questions?
- မေးစရာရှိပါသလား။

1. Give money to participants (200 each)

တစ်ယောက်ကို ဘတ် ၂၀၀ စီပေးမယ်

1. Remind participants of date and time for next workshop and call me if anything comes up (number on information sheet).

နောက်ထပ်အလုပ်ရုံဆွေးနွေးပွဲအတွက် ရက်နဲ့အချိန်ကို သတိပေးမယ်။

တစ်ခုခုဖြစ်ခဲ့လို့ရှိရင် ကျမကို ဖုန်းခေါ်လို့ရပါတယ်( ဖုန်းနံပါတ်က

သတင်းအချက်အလက်စာရွက်မှာရှိပါတယ်)

**Day 3 တတိယနေ့**

***Materials:*** *Snacks, printed photos, 200 Bath per participant, homework activity printed out, cardboard for active listening activity*

*ပစ္စည်းပစ္စယများ-မုန့်၊ ပရင့်ထုတ်ထားတဲ့ဓာတ်ပုံများ၊ တစ်ဦး ဘတ် ၂၀၀၊ ပရင့်ထုတ်ထားတဲ့ အိမ်စာများ၊ တက်တက်ကြွကြွနားထောင်စေခြင်း လုပ်ဆောင်ချက်အတွက် ကဒ်ဘုတ်တစ်ခု*

1. Ice breaker: Choose a photo that fits your mood today (from provided photo selection). အဘိုးအဘွားတို့ရဲ့ ဒီနေ့စိတ်ခံစားချက်နဲ့ကိုက်ညီမယ် ဓာတ်ပုံတစ်ပုံကို ရွေးပေးပါ( ရွေးချယ်ဖို့ပေးထားတဲ့ပုံတွေထဲမှ)

- What do you see on the photo? Why did you choose it?
- ဒီဓာတ်ပုံမှာဘာတွေ့လဲ၊ ဘာကြောင့်ဒီဓာတ်ပုံကိုရွေးရတာလဲ။
- How do you feel today? How was your week?
- ဒီနေ့မှာ ဘယ်လိုခံစားရလဲ။ ဒီတပတ်ထဲမှာရော ဘယ်လိုနေလဲ အဆင်ပြေရဲ့လား။
- Motivation to participate
- ဘာလို့ ဒီပွဲမှာပါဝင်ဖြစ်သွားတာလဲ။

1. Reflection on past week: Were there any ethical or technical issues taking photos last week?

ပြီးခဲ့တဲ့အပတ်က အတွေ့အကြုံများကို ပြန်လည်သုံးသပ်ခြင်း- စောင့်ထိန်းရမည့် ကျင့်ဝတ် ပိုင်းဆိုင်ရာကိစ္စရပ်များနှင့်ပတ်သက်ပြီး ပြဿနာတစ်ခုခုရှိခဲ့လား။

1. Activity in small groups (3-4 people): In your group, identify 3 things that you have in common and create 3 collaborative photographs to show this. Take 20 minutes for the activity.

လူ ၃-၄ယောက်ပါတဲ့ အဖွဲ့လေးများဖြင့် လုပ်ဆောင်ခြင်း-အဘိုး/အဘွားတို့အဖွဲ့မှာ ပါဝင်နေတဲ့သူတွေရဲ့ ဘုံတူညီတဲ့ အရာ ၃ ခုကိုသတ်မှတ်ပါ၊ ပြီးရင် ထိုအရာကို ပြဖို့ ဓာတ်ပုံ၃ပုံ ဖန်တီးပါ။

- Discuss in group: Think about your lives, your daily activities, your beliefs, your feelings, your culture, your relationships, what’s important to you, your favorite things or people, and the care you provide to others. What of these things do you all share?
- အဖွဲ့တွင်းဆွေးနွေးခြင်း-အဘိုး/အဘွားတို့ရဲ့ ဘဝအကြောင်း၊ နေ့စဥ်လုပ်ငန်းဆောင်တာ များအကြောင်း၊ မိမိ ၏ကိုးကွယ်ယုံကြည်မှုအကြောင်း၊ မိမိ၏ခံစားချက်များအကြောင်း၊ မိမိ ယဥ်ကျေးမှုများအကြောင်း၊ မိမိနှင့် အခြားသူများကြား ဆက်ဆံရေးအကြောင်း ၊ မိမိအတွက် အရေးကြီးတဲ့ အရာ များအကြောင်း၊ မိမိချစ်ခင်နှစ်သက်သည့်အရာများ (သို့) လူများအကြောင်းနှင့် အခြားသူများအား ပြုစုစောင့်ရှောက်မှုအကြောင်း စသည်တို့အား စဥ်းစားကြည့်ပါ။ ထိုအရာတွေနဲ့ပတ်သက်ပြီး အဘိုး/အဘွားတို့အနေနဲ့ ဘာတွေပြန်လည်မျှဝေပေးနိုင်ည မလဲ။

*Dinner (while Samia loads photos from small group activity and ‘a day in my life’ to computer) ညနေစာစားခြင်း (ဆမ်မီယာသည် အုပ်စုဖွဲ့လုပ်ဆောင်ချက်မှ ရရှိလာတဲ့ ဓာတ်ပုံများနှင့် ‘ဘဝထဲက နေ့တစ်နေ့’ မှ ဓာတ်ပုံများအား ကွန်ပျူတာထဲသို့ ထည့်သွင်းနေမယ်)*

- Each group presents their 3 collaborative photographs.
- တစ်ဖွဲ့ချင်းစီမှ ၎င်းတို့၏ ဓာတ်ပုံ ၃ ပုံအား တင်ပြရှင်းလင်းမယ်

1. Activity “good listening”: When we present photos and stories in our group, it is important that we listen to what is being said, to respect the person and be able to discuss and reflect on their experience.

“နားထောင်သူကောင်း”ဖြစ်ဖို့ လုပ်ဆောင်ခြင်း- ဓာတ်ပုံတွေနဲ့ ဇာတ်ကြောင်းတွေကို မိမိတို့အဖွဲ့ထဲမှာ တင်ပြတဲ့အခါ နားထောင်ဖို့၊ တစ်ယောက်နဲ့တစ်ယောက် လေးစားဖို့၊ အပြန်အလှန်ဆွေးနွေးဖို့၊ အတွေ့အကြုံတွေကို ပြန်လည်သုံးသပ်ဖို့ အရေးကြီးပါတယ်။

- Group: What defines a good listener? What defines a bad listener? *(write answers on card board)*
- *အဖွဲ့အလိုက်လုပ်ဆောင်ခြင်း- နားထောင်သူကောင်းဖြစ်ဖို့ ဘယ်အရာတွေက သတ်မှတ်ပေးလဲ။(ကဒ်ဘုတ်ပေါ်မှာ အဖြေများကို ချရေးပါ)*
- In pairs (2 people): Person 1 chooses a photo they took and describes it to Person 2 without showing them the photo. Person 1 tells them how they feel about the photo and what memories this photo brings up in them. Person 2 tries to actively listen without interrupting for 3 minutes.
- ၂ ယောက်တွဲပြီး လုပ်ဆောင်ခြင်း- နံပါတ်-၁လူ က သူတို့တွေရိုက်ခဲ့တဲ့ ပုံတစ်ပုံကို ရွေးလိုက်ပြီး အခြားသူတွေကိုမပြဘဲ နံပါတ်-၂လူ ကို ပြမယ်။ နံပါတ်-၁လူ က ဒီပုံနဲ့ပတ်သက်ပြီး သူတို့ဘယ်လိုခံစား ရလဲဆိုတာ ပြောပြမယ်။ ပုံက ဘယ်လိုအမှတ်တရတွေကို ယူဆောင်လာတယ် ဆိုတာကိုလည်း ပြောပြမယ်။ နံပါတ်-၂လူက ၃ မိနစ်ကြာ ဝင်ရောက်နှောင့်ယှက်ခြင်းမရှိဘဲ သေသေချာချာ နားထောင်ဖို့ကြိုးစားမယ်။
- Group: Person 2 now presents the photo of person 1 to the group. When they are done, let Person 1 add anything they forgot.
- အုပ်စုလိုက် လုပ်ဆောင်ခြင်း- အခု နံပါတ်-၂ လူက နံပါတ်-၁လူရဲ့ ပုံကို အဖွဲ့ကို တင်ပြမယ်။ ပြီးသွားပြီဆို နံပါတ်-၁လူကို မေ့ကျန်ခဲ့တာတွေကို ထပ်ဖြည့်ခိုင်းမယ်။
- Repeat the process with switched roles (now the partner is Person 1 and can choose and the photo).
- ထိုလုပ်ငန်းစဥ်ကို လူတွေလှည့်ပြီး လုပ်သွားမယ်(ခုန လက်တွဲလုပ်ဆောင်ခဲ့တဲ့ သူက နံပါတ်-၁ ဖြစ်သွားပြီး ပုံရွေးနိုင်ပါတယ်)
- Leave room for discussion – what did they find interesting? Do they think this activity will help them feel listened to in the group or become a better listener?
- ဆွေးနွေးဖို့အတွက် အခန်းထဲကနေ ထွက်ကြမယ်-ဘာတွေ စိတ်ဝင်စားစရာ တွေ့ခဲ့ လဲ။ အဖွဲ့ထဲမှာ နားထောင်ဖို့ခံစားချက်ဖြစ်လာစေရန် (သို့မဟုတ်) နားထောင် သူ ကောင်းဖြစ်လာစေရန် အခုလုပ်ဆောင်ချက်က အကူအညီဖြစ်တယ်လို့ ထင်ပါ သလား။

1. Present ‘a day in my life’ photos: “ဘဝထဲက နေ့တစ်နေ့” ဓာတ်ပုံများအား တင်ပြခြင်း

- Let every person stand up and present their series ‘a day in my life’ with the photos and explanation.
- လူတိုင်းကို မတ်တပ်ရပ်စေပြီး သူတို့ရဲ့ “ဘဝထဲက နေ့တစ်နေ့” ဓာတ်ပုံစီးရီးအား ရှင်းလင်းတင်ပြစေမယ်။
- Are there common experiences of everyone’s daily life? What is different? How do people feel about seeing the other person’s perspective? Is there anything surprising or new that you did not know before?
- လူတိုင်းရဲ့ နေ့စဥ်ဘဝထဲမှာ တူညီတဲ့အတွေ့အကြုံတွေရှိလား။ မတူတာရောဘာ တွေရှိမလဲ။ အခြားသူတစ်ယောက်ရဲ့ အမြင်နဲ့ပတ်သက်ပြီး လူတွေက ဘယ်လို ခံစားကြရလဲ။ အရင်တုန်းကမိမိ မသိခဲ့တဲ့အသစ်တွေ (သို့မဟုတ်) အံ့သြစရာတွေ ရှိသလား။
- Discuss experience of taking photos for this activity. Was it fun? Was it difficult?
- ယခုလုပ်ဆောင်ချက်အတွက် ဓာတ်ပုံရိုက်ကူးခဲ့တဲ့ အတွေ့အကြုံကို ဆွေးနွေးပေးပါ။ ပျော်စရာကောင်းလား။ အခက်အခဲရှိခဲ့လား။

1. Homework:

Photograph 3 things that you value and want to stay the same and 3 things that you want to change. Again, think about your lives as older person in this village, your daily activities including care responsibilities, your relationships to others, and your role in your family or community.

အိမ်စာ-

မိမိ တန်ဖိုးထားသော၊ မပြောင်းလဲဘဲရှိစေချင်သော အရာ ၃ခု နှင့် ပြောင်းလဲစေချင်သော အရာ ၃ ခု ကို ဓာတ်ပုံရိုက်ခဲ့ပါ။ ဒီရွာမှာ အသက်အရွယ်ကြီးရင့်သူတစ်ဦးအဖြစ် မိမိရဲ့ ဘဝအကြောင်း၊ အခြားသူများအား ပြုစုစောင့်ရှောက်မှုအပါအဝင် မိမိ၏ နေ့စဥ်လုပ်ငန်းဆောင်တာများအကြောင်း၊မိမိနှင့် ​အခြားသူများကြား ဆက်ဆံရေး အကြောင်း ၊ မိသားစုတွင်း (သို့) ရပ်ရွာတွင်း၌ မိမိ၏ အခန်းကဏ္ဍ စသည်တို့အား စဥ်းစားကြည့်ပါ။

- Any questions?
- မေးစရာရှိပါသလား။

1. Give money to participants (200 each)

တစ်ယောက်ကို ဘတ် ၂၀၀ စီပေးမယ်

1. Remind participants of date and time for next workshop and call me if anything comes up (number on information sheet).

နောက်ထပ်အလုပ်ရုံဆွေးနွေးပွဲအတွက် ရက်နဲ့အချိန်ကို သတိပေးမယ်။

တစ်ခုခုဖြစ်ခဲ့လို့ရှိရင် ကျမကို ဖုန်းခေါ်လို့ရပါတယ်( ဖုန်းနံပါတ်က

သတင်းအချက်အလက်စာရွက်မှာရှိပါတယ်)

**Day 4 စတုတ္ထနေ့**

***Materials:*** *Snacks, 200 Bath per participant, bring printed photos that were discussed in previous sessions, homework activity printed out, carboard paper for collecting points from homework discussion, paper and pens for captioning activity, printed caption activity tasks*

*ပစ္စည်းပစ္စယများ- မုန့်၊ တစ်ယောက် ဘတ် ၂၀၀၊ အရင်ဆွေးနွေးပွဲတုန်းက ဆွေးနွေးခဲ့သော ပရင့်ထုတ်ထားတဲ့ ဓာတ်ပုံများ၊ ပရင့်ထုတ်ထားတဲ့ အိမ်စာ၊ အိမ်စာဆွေးနွေးချက်များအတွက် ပွိုင့်များ ပေးရန် ကဒ်ဘုတ်စာရွက်၊ “ခေါင်းစီးပေးခြင်း” လုပ်ဆောင်ချက်အတွက် စာရွက်နှင့် ဘောလ်ပင်များ၊ “ခေါင်းစီးပေးခြင်း” လုပ်ဆောင်ချက်အတွက် ပရင့်ထုတ်ထားသည့် ဆောင်ရွက်ချက်များ*

1. Ice breaker: Choose a photo that is meaningful to you. Describe the photo with the five questions you learned last week.

အဘိုး/အဘွားတို့အတွက် အဓိပ္ပာယ်ရှိသည့် ပုံတစ်ပုံရွေးပေးပါ။ ပြီးခဲ့တဲ့အပတ်က လေ့လာခဲ့တဲ့ မေးခွးန်ငါးခုအား သုံးပြီး ပုံကို ပြောပြပေးပါ။

- What do you see here?
- What's really happening here?
- How does this relate to our lives?
- Why does this problem, concern, or strength exist?
- What can we do about it?
- ဒီမှာ ဘာတွေ့ရလဲ။
- ဒီမှာ ဘာတွေဖြစ်နေလဲ။
- ဒီဟာနဲ့ မိမိတို့ဘဝ ဘယ်လို ဆက်စပ်နေလဲ။
- ဒီပြဿနာက (သို့မဟုတ်) ဒီကောင်းတဲ့ကိစ္စက ဘာကြောင့်ရှိနေရတာလဲ။
- ဒါနဲ့ပတ်သက်ပြီး မိမိတို့ဘာတွေလုပ်နိုင်မလဲ။

1. Reflection on past week: Were there any ethical or technical issues taking photos last week?

ပြီးခဲ့တဲ့အပတ်က အတွေ့အကြုံများကို ပြန်လည်သုံးသပ်ခြင်း- စောင့်ထိန်းရမည့် ကျင့်ဝတ် ပိုင်းဆိုင်ရာကိစ္စရပ်များနှင့်ပတ်သက်ပြီး ပြဿနာတစ်ခုခုရှိခဲ့လား။

1. Practice captioning photos: There are many ways to describe a photo and use words to share an experience. It brings a photo alive (e.g., like in a newspaper article – you see a photo and you have words to explain a story). Captions are helpful to further understand the context of a photo and not just repeat what can be seen.

ဓာတ်ပုံ ခေါင်းစီးတပ်ခြင်း- အတွေ့အကြုံတစ်ခုအား စကားလုံးသုံးပြီး ပုံတစ်ပုံနှင့် ပုံဖော်ရာ၌ နည်းလမ်းများစွာရှိပါတယ်။ စကားလုံးတွေက ပုံကို အသက်ဝင်သွားစေတယ် (ဥပမာ- သတင်းစာဆောင်းပါးတစ်ပုဒ်လိုမျိုးပေါ့- အဲ့ဒီမှာဆို ပုံတစ်ပုံကို တွေ့ရမယ် ပြီးတော့ ဇာတ်လမ်းကို ရှင်းပြပေးနေတဲ့ စကားလုံးတွေရှိမယ်)။

1. Divide the group into three small groups of 3-4 people and present each group with one captioning technique. Let them read about the technique, choose one photo and create one caption together.

လူ ၃-၄ယောက်ပါတဲ့ အဖွဲ့သေးလေးတွေ ဖွဲ့လိုက်မယ်။ တစ်ဖွဲ့ချင်းစီကို ပုံခေါင်းစီးတပ်တဲ့နည်းလမ်းတစ်ခုစီ ပြောပြမယ်။ အဆိုပါနည်းလမ်းအား အဖွဲ့တွေကို ဖတ်စေမယ်။ ပုံတစ်ပုံရွေးချယ်ပြီးတော့ ပုံခေါင်းစီးတစ်ခုကို အတူတူ ဖန်တီး ကြမယ်။

1. Write a “sense poem” about the photograph:

I hear…

I smell…

I see…

I taste…

I touch…

I feel…

Then, delete the words that are provided here so that only your words remain. Feel free to edit as you like.

ဓာတ်ပုံ နဲ့ပတ်သက်ပြီး “အာရုံခံစားမှု ကဗျာ” တစ်ပုဒ်ရေးပါ။

ငါ...............ကြားတယ်.

ငါ ..................အနံ့ရတယ်.

ငါ ....................မြင်တယ်

ငါ .....................အရသာခံမိတယ်

ငါ ............ထိတွေ့ကိုင်တွယ်တယ်

ငါ.............ခံစားရတယ်

ပြီးရင် ကိုယ်ရဲ့စာလုံးများပဲချန်ပြီး ပေးထားတဲ့ စာလုံးတွေကို ဖျက်ပစ်ပါ။ မိမိစိတ်ကြိုက် လွတ်လွတ်လပ်လပ် ပြန်ပြင်လို့လည်းရပါတယ်။

1. Write a photo story for one photograph. It can be similar to a diary entry or a short story. Answering these questions can help:

What happened 1 minute before and afterwards?

What happened 1 hour before and afterwards?

What happened 1 day before and afterwards?

ပုံတစ်ပုံအတွက် ဓာတ်ပုံဇာတ်လမ်းတစ်ခု ရေးပေးပါ။ ဒိုင်ယာရီ (သို့မဟုတ်)​ဇာတ်လမ်းတိုလေးတစ်ပုဒ် ရေးသလိုမျိုးပါပဲ။

အောက်ကမေးခွန်းတွေကို ဖြေကြည့်လိုက်ပါ၊ မိမိအတွက် အထောက်အကူဖြစ်ပါလိမ့်မယ်။

ပုံထဲက အဖြစ်အပျက် မဖြစ်ခင် ၁ မိနစ် နဲ့ ဖြစ်ပြီးနောက် ၁ မိနစ်မှာ ဘာတွေဖြစ်ခဲ့လဲ။

ပုံထဲက အဖြစ်အပျက် မဖြစ်ခင် ၁ နာရီ နဲ့ ဖြစ်ပြီးနောက် ၁ နာရီမှာ ဘာတွေဖြစ်ခဲ့လဲ။

ပုံထဲက အဖြစ်အပျက် မဖြစ်ခင် ၁ ရက် နဲ့ ဖြစ်ပြီးနောက် ၁ ရက်မှာ ဘာတွေဖြစ်ခဲ့လဲ။

1. Use the following six questions as a start to describe what the photo means:

When? Where? Who? What? How? Why?

Then, think of any words or ideas that come to your mind when you look at the photo and write them down. They do not need to make sense or be related.

ပုံက ဘာကို ဆိုလိုတာလဲဆိုတာကို စပြောဖို့အတွက် အောက်က မေးခွန်း ၆ ခုကို အသုံးပြုပါ။

ဘယ်အချိန်လဲ? ဘယ်နေရာလဲ? ဘယ်သူလဲ? ဘာအကြောင်းလဲ? ဘယ်လိုလဲ? ဘာကြောင့်လဲ?

ပြီးရင် ပုံကိုကြည့်လိုက်တဲ့အချိန် ကိုယ်စိတ်ထဲပေါ်လာတဲ့ စကားလုံး (သို့မဟုတ်) အတွေးကို ရေးချလိုက်ပါ။ အဓိပ္ပာ​ယ်ရှိစရာမလိုဘူး၊ အကျိုးအကြောင်း ဆက်စပ်စရာလည်းမလိုဘူး။

1. Group: Let each group present the technique they learned to the others, using a photo and caption as an example.

အဖွဲ့လိုက်လုပ်ဆောင်ခြင်း- နမူနာ ပုံတစ်ပုံ နဲ့ ခေါင်းစီးတစ်ခုကိုအသုံးပြုပြီး တစ်ဖွဲ့ချင်းသည် မိမိတို့လေ့လာခဲ့တဲ့ နည်းစနစ်ကို အခြားသူများကို ပြောပြ မယ်။

1. To have an exhibition, one needs a main message. You have all discussed your photos and experiences over the past weeks. Although everyone has a unique perspective, there were some themes that you all shared and discussed: caregiving, nature, and religion. Before we think about the main message, it will be helpful to use the photos that were discussed and caption them using your your previous discussions that Samia printed out for you.

၁။ ဓာတ်ပုံပြပွဲတခုပြုလုပ်ရန်အတွက် အခရာကျသော သတင်းပေးမှု လိုအပ်သည်။  လွန်ခဲ့သော ရက်သတ္တပတ်များအတွင်း၌ သင့်ဓာတ်ပုံများနှင့် အတွေ့အကြုံအားလုံးကို သင်ကိုယ်တိုင် ဆွေးနွေးထားပြီးဖြစ်လေသည်။  လူတိုင်းတွင် ထူးခြားသောအမြင်ရှိသော်လည်း ဆွေးနွေးခဲ့သောအကြောင်းအရာများ၌ ပြုစုစောင့်ရှောင့်ပေးခြင်း၊ ကိုးကွယ်ယုံကြည်မှုနှင့် ပင်ကိုယ်သဘာဝစရိုက်လက္ခာဏာ စသည်တို့တွင် ယေဘုယျတူညီမှုညီကြလေသည်။  အဓိကသတင်းအချက်အလက်ပေး မစဉ်းစားမီ၊ ဆွေးနွေးထားပြီဖြစ်သော ဓာတ်ပုံများကို ထည့်သွင်းသုံးသပ်ခြင်းနှင့် သင်၏ယခင်ဆွေးနွေးမှုများကို အသုံးပြုခြင်းတို့သည် ၎င်းတို့ကို စာတန်းထိုးရန် အထောက်အကူဖြစ်ပါမည်။ အဆိုပါ ဓာတ်ပုံနှင့် ဆွေးနွေးမှုများသည် သင့်အတွက် ကြှနျုပျတို့ ထုတ်ဝေပေးထားပြီးဖြစ်လေသည်။

1. Divide the group into three small groups of 3-4 people and let each group choose a topic (care, nature, religion).

(၁) အဖွဲ့ကို လူ ၃ ဦး (သို့မဟုတ်) ၄ ဦး စီရှိသော အုပ်စုငယ်သုံးစုခွဲပြီး အုပ်စုတစ်ခုစီအား ခေါင်းစဉ်တစ်ခု (ပြုစုစောင့်ရှောင့်ပေးခြင်း၊ ကိုးကွယ်ယုံကြည်မှုနှင့် ပင်ကိုယ်သဘာဝစရိုက်လက္ခာ) စသည်တို့အနက်မှ တခုခုကို ရွေးချယ်စေပါ။

1. Let every group choose photos they want to present and match them with captions provided. Feel free to edit these accordingly.

(၂) အဖွဲ့တိုင်းကို တင်ပြလိုသော ဓာတ်ပုံများကို ရွေးချယ်ပြီး ပေးထားသည့် စာတန်းများနှင့် ကိုက်ညီပါစေ။  ပြီးနောက် မိမိစိတ်တိုင်းကျ လိုအပ်သလို ပြင်ဆင်ပါ။

1. Think about the overall message of this theme. What do you want to share with other people? What could be problems? What do you want to suggest to others to change this problem or raise awareness for it?

(၃) ဤအကြောင်းအရာ၏ အလုံးစုံသော အဓိကပင်မ သတင်းအချက်အလက်ခေါင်းစဥ်ကို စဉ်းစားပါ။  တခြားလူတွေကို ဘာတွေမျှဝေချင်သလဲ။  ဘယ်အရာတွေက ပြဿနာ ဖြစ်နိုင်လဲ။  ဤပြဿနာကို ပြောင်းလဲရန် သို့မဟုတ် သတိပြုမိစေရန် အခြားသူများအား သင်ဘာကို အကြံပြုလိုသလဲ။

1. Each small group presents their photos and captions to the group.

(၄) အဖွဲ့ငယ်တစ်ခုစီသည် ၎င်းတို့၏ဓာတ်ပုံများနှင့် စာတန်းများကို အဖွဲ့အား တင်ပြရမည်ဖြစ်သည်။

*Dinner (while Samia loads photos from last homework to computer) ညနေစာ (ဆမ်မီယာက အရင်တပတ်အိမ်စာဓာတ်ပုံတွေကို ကွန်ပျူတာထဲသို့ ထည့်နေမည်)*

1. Group activity: Present photos from last homework on what to change and what to keep the same.

- Let every person stand up and present their 3 photos they want to keep the same.
- Are there common experiences of what people value and want to keep the same? *(Collect notes on cardboard paper and on Canva* [*https://www.canva.com/design/DAFZ30JY7ns/CzGWHntkAw6XWOjTahYLUg/edit?utm_content=DAFZ30JY7ns&utm_campaign=designshare&utm_medium=link2&utm_source=sharebutton*](https://www.canva.com/design/DAFZ30JY7ns/CzGWHntkAw6XWOjTahYLUg/edit?utm_content=DAFZ30JY7ns&utm_campaign=designshare&utm_medium=link2&utm_source=sharebutton) *)*
- Then, let every person stand up and present their 3 photos of things they want to change.
- Why do people want to change this? What is the underlying theme of the issues people want to change? *(Collect notes on cardboard paper)*
- Discuss how these changes could be achieved. What are potential (realistic) solutions that one could suggest to policymakers or the community?
- Think about how a photo exhibition could help raise awareness for the issues identified and help implement and suggest these solutions. Would participants want to share their photos (anonymously) in an exhibition to elicit changes?

အဖွဲ့လိုက် လုပ်ဆောင်ချက်- အရင်တပတ် အိမ်စာက ပုံတွေထဲမှာ ဘာတွေပြောင်းလဲ စေချင်လဲ၊ ဘာတွေကို မပြောင်းလဲဘဲထားချင်လဲဆိုတာကို ပြောပြကြမယ်။

- တစ်ယောက်ချင်းစီကို မတ်တပ်ရပ်စေပြီး သူတို့ မပြောင်းလဲဘဲထားချင်တဲ့ ပုံ ၃ ပုံကို ပြောပြရပါမယ်။
- ဘာတွေကို တန်ဖိုးထားပြီး၊ ဘာတွေကို မပြောင်းလဲဘဲ ထားချင်လဲဆိုတာနဲ့ ပတ်သက်ပြီး ဘုံတူညီတဲ့အချက်တွေရှိလား။
- ပြီးတော့ တစ်ယောက်ချင်းစီကို မတ်တပ်ရပ်စေပြီး သူတို့မပြောင်းလဲစေချင်တဲ့ ပုံ ၃ ပုံကို ပြောပြရပါမယ်။
- ဘာကြောင့်ပြောင်းလဲစေချင်တာလဲ။ ပြောင်းလဲစေချင်တဲ့ နောက်ကွယ်က အကြောင်းက ဘာလဲ။ (ကဒ်ဘုတ်စာရွက်ပေါ်မှာ မှတ်စုတွေကို စုစည်းမယ်)
- ဘယ်လိုပြောင်းလဲနိုင်မလဲ​ ဆွေးနွေးပေးပါ။ မိမိတို့အနေဖြင့် မူဝါဒချမှတ်သူများ (သို့မဟုတ်) ရပ်ရွာကို လက်တွေ့ကျတဲ့ ဘယ်လိုဖြေရှင်းနည်းတွေကို အကြံပေးချင်လဲ။
- ဒီပြဿနာနဲ့ပတ်သက်ပြီး လူတွေသတိမူမိအောင်၊ ဖြေရှင်းနည်းတွေကို အကြံပေးအကောင်အထည်ဖော်နိုင်အောင် ဓာတ်ပုံပြပွဲကနေ ဘယ်လိုတွေ ကူညီပေးလို့ရနိုင်မလဲဆိုတာ စဥ်းစားကြည့်ပေးပါ။ မိမိတို့ ပြောင်းလဲစေချင်သည်များ ပြောင်းလဲရန်အတွက် ဓာတ်ပုံတွေကို အမည်ဝှက်၍ ပြပွဲတွင် မျှဝေချင်ပါသလား။

1. Discussion: Would you be interested in showing these photos to your community or other people in the world in an exhibition?

If no, why? Discuss issues. (Leave room to let participants discuss)

ဆွေးနွေးချက်- ဓာတ်ပုံပြပွဲတစ်ခုခုတွင် သင့်အသိုင်းအဝိုင်း သို့မဟုတ် ကမ္ဘာပေါ်ရှိ အခြားလူများအား ဤဓာတ်ပုံများကို ပြသရန် သင်စိတ်ဝင်စားမှုရှိပါသလား။

 မရှိရင် ဘာကြောင့် မရှိရတာပါလဲ။

1. Arrange a time and date for one-on-one interviews, where we will discuss up to three photos and caption them together.

ပုံ ၃ ပုံနှင့် ခေါင်းစီးများကို အတူတူ ဆွေးနွေးဖို့ရန် တစ်ဦးချင်း အင်တာဗျူးအတွက် ရက်နှင့် အချိန်အား စီစဥ်မည်။

1. Homework:

အိမ်စာ

In preparation for the interview: Think about the following questions before and after you take the photos and take notes / try to caption three photos you want to share on this topic: Do you feel valued and respected as an older person in your community? Do you feel like your care contributions are seen and appreciated by others? What would need to change in order for you and other older people to feel respected, valued, and appreciated?

အင်တာဗျူးအတွက် ပြင်ဆင်ခြင်း- မိမိမျှဝေချင်သော ပုံ ၃ ပုံအား မရိုက်ခင် နှင့် ရိုက်ပြီး၊ မှတ်စုမယူခင်နှင့် ယူပြီး၊ ခေါင်းစီး မပေးခင်နှင့် ပေးပြီးနောက်တွင် အောက်ပါ မေးခွန်းများကို စဥ်းစားပေးပါ။

သင့်ရပ်ရွာထဲတွင် အဘိုးအဘွားတစ်ဦးအနေဖြင့် တန်ဖိုးထား လေးစားခံရ တယ်လို့ ခံစားရသလား။ သင့်ရဲ့ ကူညီပြုစုစောင့်ရှောက်မှုအား တခြားသူတွေအနေနဲ့ သိမြင် အသိအမှတ်ပြုတယ်လို့ ခံစားရသလား။ သင်နှင့် အခြားအဘိုးအဘွားများကို လေးစားတန်ဖိုးထား အသိအမှတ်ပြုဖို့အတွက် ဘာတွေပြောင်းလဲဖို့လိုသလဲ။

- Any questions?
- မေးစရာရှိပါသလား

1. Give money to participants (200 each)

တစ်ယောက်ကို ဘတ် ၂၀၀ စီပေးမယ်

1. Remind participants of date and time for interviews and next workshop and call me if anything comes up (number on information sheet).

အင်တာဗျူးနှင့် နောက်ထပ်အလုပ်ရုံဆွေးနွေးပွဲအတွက် ရက်နဲ့အချိန်ကို သတိပေးမယ်။

တစ်ခုခုဖြစ်ခဲ့လို့ရှိရင် ကျမကို ဖုန်းခေါ်လို့ရပါတယ်( ဖုန်းနံပါတ်က

သတင်းအချက်အလက်စာရွက်မှာရှိပါတယ်)

**Day 5**

**၅ ရက်မြောက်နေ့**

***Materials:*** *Snacks, 200 Bath per participant, prepared photos to showcase*

*ပစ္စည်းပစ္စများ- မုန့်၊ တစ်ယောက် ဘတ် ၂၀၀စီ၊ ပြပွဲအတွက်ပြင်ဆင်ထားသည့် ဓာတ်ပုံများ*

1. Were you able to get consent for photos that include faces?

မျက်နှာပါတဲ့ ပုံတွေအတွက် သသောတူခွင့်ပြုချက်ယူနိုင်ခဲ့ပါသလား

1. Let everyone present their 3 photos they discussed and captioned in the interviews to the group.

အင်တာဗျူးတွင် ဆွေးနွေးပြီး ခေါင်းစဥ်တပ်ခဲ့သောပုံ ၃ ပုံအား တစ်ယောက်ချင်းစီမှ တင်ပြပေးပါ။

1. We need a plan for the exhibition to have it next week: When? Where? Who to invite? How will we arrange the photos?

နောက်တပတ်မှာပြုလုပ်မည့်ပြပွဲအတွက် အစီအစဥ်တစ်ခုလိုအပ်ပါတယ်- အချိန်၊ နေရာ၊ ဖိတ်မယ့်သူ။ ကျမတို့တွေ ပုံတွေကို ဘယ်လိုတွေ နေရာချကြမလဲ။

- We should meet at the location earlier that day to hang the photos.
- ကျမတို့တွေ ဓာတ်ပုံတွေကိုချိတ်ဖို့ ပြပွဲလုပ်မယ့်နေရာမှာ တစ်ရက်ကြိုပြီး ဆုံဖို့လိုပါမယ်ရှင်
- Ben Lu and Samia will present the project, does anyone else want to say something at the event?
- အင်ဒူးနဲ့ ကျမက စီမံကိန်းနဲ့ပတ်သက်ပြီး ပြောပါမယ်။ တခြားအဘိုးအဘွားတို့ရော ပွဲမှာ တစ်ခုခုပြောချင်တာများရှိလားရှင်။

1. Select date, time, and location and remind participants to invite their friends and family and that we will meet again after the exhibition **on March 4** to ask for feedback and finish the project.

နေ့ရက်၊​အချိန်နဲ့ နေရာရွေးချယ်မယ်။ စီမံကိန်းမှာပါဝင်ခဲ့တဲ့အဘိုးအဘွားတွေကို သူတို့ရဲ့ သူငယ်ချင်းတွေ၊ မိသားစုတွေကို ဖိတ်ဖို့ သတိပေးပါမယ်။ ပြပွဲပြီးနောက် **မတ်လ ၄ ရက်နေ့မှာ** ဝေဖန်အကြံပြုချက်တွေအတွက် ပြန်ဆုံပါမယ်။ ပြီးရင် စီမံကိန်းပြီးပါပြီ။

1. Give money to participants (200 each)

တစ်ယောက်ကို ဘတ် ၂၀၀ စီပေးမယ်
